# Spike structure of gold nanobranches induces hepatotoxicity in mouse hepatocyte organoid models

**DOI:** 10.1186/s12951-024-02363-1

**Published:** 2024-03-05

**Authors:** Rui Zhang, Dan Li, Ruibo Zhao, Dandan Luo, Yeting Hu, Shengyan Wang, Xiaolu Zhuo, M. Zubair Iqbal, Han Zhang, Qianqian Han, Xiangdong Kong

**Affiliations:** 1 Institute for Smart Biomedical Materials, School of Materials Science & Engineering, Zhejiang Sci-Tech University, Hangzhou, 310000 PR China; 2 Zhejiang-Mauritius Joint Research Center for Biomaterials and Tissue Engineering, Zhejiang Sci-Tech University, Hangzhou, 310018 PR China; 3 Department of Colorectal Surgery and Oncology, Key Laboratory of Cancer Prevention and Intervention, The Second Affiliated Hospital, Ministry of Education, Zhejiang University School of Medicine, Hangzhou, 310030 PR China; 4 School of Science Engineering, The Chinese University of Hong Kong, Shenzhen, Guangdong 518172 PR China; 5 National Institutes for Food and Drug Control, Beijing, PR China

**Keywords:** Gold nanobranches, Hepatocyte organoid, Spike structure, Hepatotoxicity, Toxicology screening platform

## Abstract

**Background:**

Gold nanoparticles (GNPs) have been extensively recognized as an active candidate for a large variety of biomedical applications. However, the clinical conversion of specific types of GNPs has been hindered due to their potential liver toxicity. The origin of their hepatotoxicity and the underlying key factors are still ambiguous. Because the size, shape, and surfactant of GNPs all affect their properties and cytotoxicity. An effective and sensitive platform that can provide deep insights into the cause of GNPs’ hepatotoxicity in vitro is therefore highly desired.

**Methods:**

Here, hepatocyte organoid models (Hep-orgs) were constructed to evaluate the shape-dependent hepatotoxicity of GNPs. Two types of GNPs with different nanomorphology, gold nanospheres (GNSs) and spiny gold nanobranches (GNBs), were synthesized as the representative samples. Their shape-dependent effects on mice Hep-orgs’ morphology, cellular cytoskeletal structure, mitochondrial structure, oxidative stress, and metabolism were carefully investigated.

**Results:**

The results showed that GNBs with higher spikiness and tip curvature exhibited more significant cytotoxicity compared to the rounded GNSs. The spike structure of GNBs leads to a mitochondrial damage, oxidative stress, and metabolic disorder in Hep-orgs. Meanwhile, similar trends can be observed in HepG2 cells and mice models, demonstrating the reliability of the Hep-orgs.

**Conclusions:**

Hep-orgs can serve as an effective platform for exploring the interactions between GNPs and liver cells in a 3D perspective, filling the gap between 2D cell models and animal models. This work further revealed that organoids can be used as an indispensable tool to rapidly screen and explore the toxic mechanism of nanomaterials before considering their biomedical functionalities.

**Supplementary Information:**

The online version contains supplementary material available at 10.1186/s12951-024-02363-1.

## Introduction

Gold nanoparticles (GNPs) are one of the promising nanomaterials used in the field of bio-applications, such as targeted drug delivery, molecular diagnostics, bioimaging, and cancer phototherapy [1–5]. With the latest advancements in synthetic and manufacturing technologies, the composition, size, shape, surface charge, and surfactant chemistry of GNPs can be precisely manipulated [6, 7], endowing them with various biomedical functions. However, the clinical translation of GNPs has been limited primarily due to their hepatotoxicity. Although coating GNPs with biocompatible molecules can improve their biocompatibility to some extent [8], the unclear toxic mechanism of GNPs is still a main obstacle in their way to clinical applications and regulatory approval [9].

Previous studies have suggested that the shape of GNPs can significantly affect their properties along with their cellular uptake and accumulation behaviors, leading to a difference in their cytotoxicity [10, 11]. For example, the nanomorphology of the nanoparticles (NPs) can affect their cell uptake behavior. When the surface turned sharp, a 4.6-fold increase in cell uptake was observed compared to the rounded one [12]. Meanwhile, non-spherical NPs are expected to have a higher cell-targeting specificity than the spherical counterparts, suggesting a higher toxicity of non-spherical NPs [13]. Studies have also shown that spiky nanostructures could exert mechanical stress on cells, leading to potassium efflux and activation of inflammasomes during the internalization process [14]. In addition, needle-shaped and plate-shaped NPs induced the most significant cell death and IL-6 outbreak compared to the spherical and rod-shaped ones [15]. The shape-mediated toxicity might be positively correlated with factors such as specific surface area, conductivity, and surface charge [16]. Crystals with large specific surface area and sharp tips can easily scratch the cell membrane, causing cell apoptosis, crystal aggregation and retention in the liver and kidneys [17]. Further investigation and in-depth analysis are still needed to clarify the shape-dependent toxicity of GNPs. It is urgent to push the development of in vitro biological toxicity screening models to accelerate the clinical translation of nanomaterials [18].

Organoid models can provide a three-dimensional view, similar internal microenvironment, and strong intercellular connections, holding promise to overcome the limitations of traditional cell models for evaluating nanoparticle safety and efficacy of nanomaterials [19, 20]. In recent years, many studies have used organoid models to evaluate the safety of NPs. For example, a mouse kidney organoid models has been constructed to screen the renal toxicity of quantum dots. The results have demonstrated that black phosphorus quantum dots (BP-QDs) could enter and accumulate in the kidney, causing severe adverse effects [21]. The renal toxicity of BP-QDs has been further validated in mice and human cells. This is the first report using organoid models as the platform for the toxicity evaluation of inorganic NPs. Next, Yan Huang et al. cultivated brain organoids from induced pluripotent stem cells to assess the impact of silver NPs exposure on the brain tissue [22]. Ag NPs at low concentrations (0.1 µg mL^–1^) could promote cell proliferation and inhibit neuronal apoptosis. Meanwhile, they could affect cilia assembly and elongation, disrupt the cell cycle, and cause abnormal growth of brain organoids. Conversely, at high concentrations (0.5 µg mL^–1^), Ag NPs could inhibit astrocyte differentiation and promote neuronal apoptosis. This confirms that brain organoids can serve as a new platform for studying neurotoxicity and cellular mechanisms of nanomaterials. Furthermore, brain organoids expressing cortical proteins have been used to study the neurotoxicity induced by ZnO NPs. The results demonstrated that high concentrations (64 µg mL^–1^) of ZnO NPs exhibit cytotoxicity on brain organoids through defective autophagy and intracellular accumulation of zinc ions [23]. These studies all demonstrate that organoid model is a powerful platform for evaluating the safety and efficacy of NPs in vitro.

A previous research has reported the shape-dependent toxicity of GNPs against osteoblast and osteosarcoma in vitro models [24]. However, due to the preferential accumulation and metabolism of GNPs in the liver [25, 26], the study on the hepatotoxic effects of different shaped NPs with similar particle size is necessary. Hep-orgs, retaining key morphological, functional, and gene expression characteristics of the liver, can be generated from individual liver cells and cultured in vitro for several months [27–29], therefore can serve as an in vitro model to study the shape-dependent hepatotoxicity of GNPs from a 3D perspective at the level of microtissues, cells, and molecules. The circulation between cells simulates the actual transport of NPs in the organism, the extensive cell-cell, the cell-matrix interactions, as well as the organ-like structure and morphology, mimicking the physiological tissue microenvironment. This provides an opportunity to screen and study the hepatotoxicity and metabolism of GNPs.

**Scheme 1:**
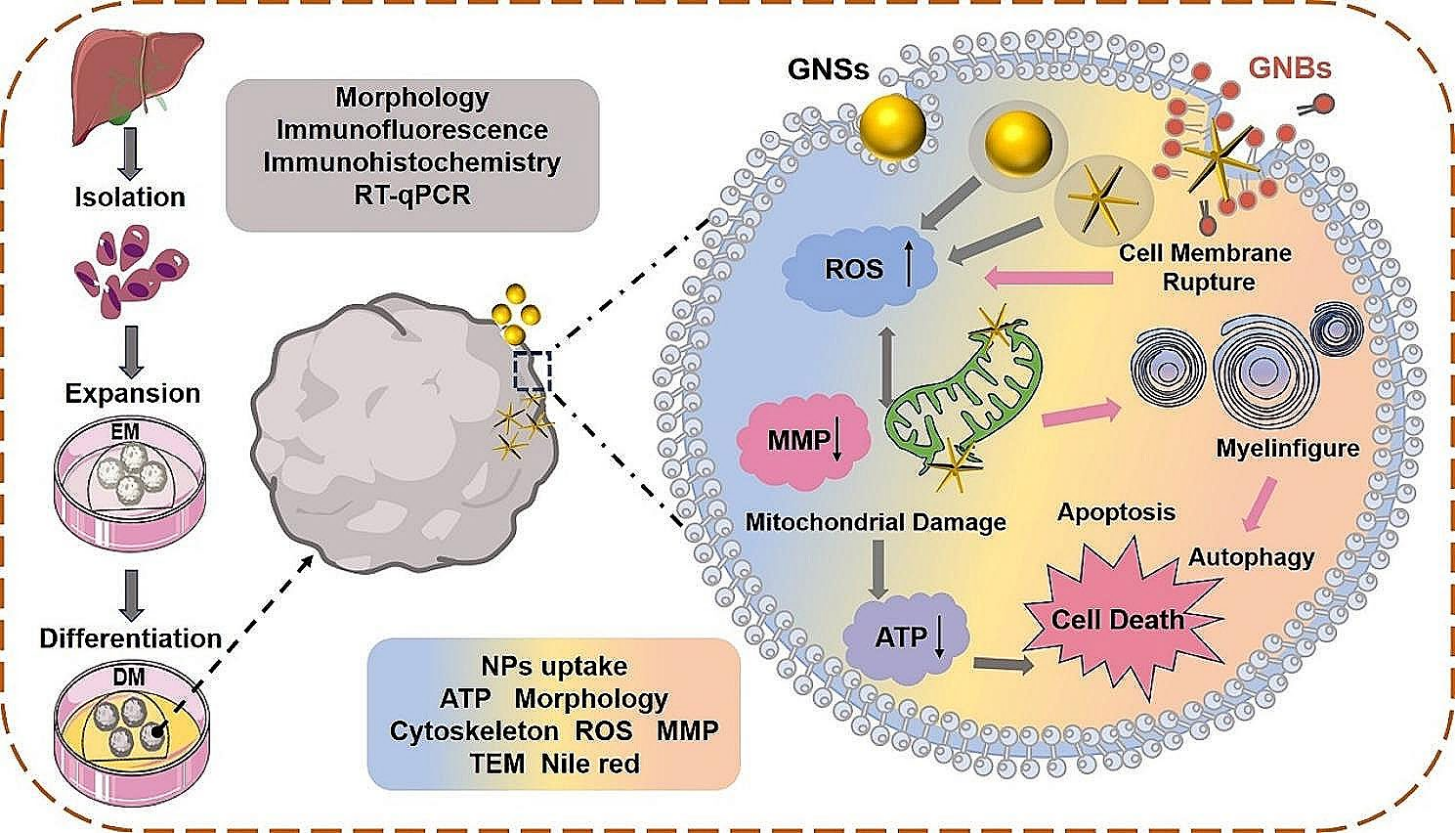
Schematic diagram of the generation of Hep-orgs, evaluation process of GNPs’ hepatotoxicity, and the plausible mechanisms underlying the toxicity

In this work, we synthesized two types of GNPs, gold nanospheres (GNSs) and spiny gold nanobranches (GNBs), through the seed-mediated method. Their shape-dependent hepatotoxicity was carefully studied by the Hep-orgs (Scheme 1). The cellular morphology, cytoskeletal structure, mitochondrial structure, reactive oxygen species (ROS) generation, and lipid metabolism in Hep-orgs were comprehensively investigated. The morphological and structural changes of intracellular organelles in liver cells were observed through the biological transmission electron microscopy (TEM). We also validated the hepatotoxicity of GNPs at both cellular and animal levels. This work confirms that GNBs with nano spikes increase oxidative stress and metabolic abnormalities by damaging mitochondria in Hep-orgs. Our research provides an effective approach for the assessment of their hepatotoxicity from a 3D perspective in vitro.

## Materials and methods

### Materials

Chloroauric acid (HAuCl_4_·4H_2_O), cetyltrimethylammonium bromide (CTAB), cetyltrimethylammonium chloride (CTAC), ascorbic acid (AA), sodium borohydride (NaBH_4_), silver nitrate (AgNO_3_), and triton X-100 (TX) were purchased from Aladdin. Dulbecco’s modified eagle Medium (DMEM), DMEM/F12, fetal bovine serum (FBS), 4′,6-diamidino-2-phenylindole (DAPI) were purchased from Gibco (USA). CellTiter-Glo 3D Cell Viability were purchased from Sigma (USA). Trypsin-EDTA, penicillin/streptomycin, ROS assay kit, and mitochondrial membrane potential assay kit (JC-1) were purchased from Beyotime Inc. (Shanghai, China). All antibodies come from Abcam (UK). All cell growth factors come from Novoprotein (Shanghai, China). Deionized water prepared by the Milli-Q system (Millipore, USA) was used in all the experiments, and all consumables and Matrigel were purchased from Corning (USA).

### Cell and organoid culture

293t-HA-Rspon1-Fc cells were used as previously described to generate conditional medium and methods for R-spondin1 [30]. HepG2 (Human hepatoma cell line) cells were purchased from American Type Culture Collection. HepG2 cells were cultured in DMEM medium with 10% FBS and 1% penicillin (100 U/mL)/streptomycin. Cells at 80-90% confluence were detached using 0.25% Trypsin-EDTA and subcultured onto a new dish. All cells were cultured at 37℃ in a humidified atmosphere with 5% CO_2_.

The isolation and culture methods of mice hepatocyte organoid refer to a published paper [27–29]. Specifically, the method is as follows, C57BL/6 mice were euthanized by cervical dislocation and immersed in 75% ethanol for disinfection. Under sterile conditions, the liver tissue from the mice was isolated in pre-chilled PBS containing 1% penicillin and streptomycin. The tissue was washed with washing medium 2–3 times to remove impurities and red blood cells as much as possible. The washed liver tissue was transferred to a new dish and cut into fragments of approximately 1–2 mm^3^. The fragmented tissue was collected in a centrifuge tube, centrifuged at 1100 rpm for 3 min, and the supernatant was discarded. An appropriate amount of digestion solution (containing 0.125 mg/mL Collagenase IV and Dispase II) was added according to the tissue volume, and the tissue was shaken and digested at 37℃ for 30 min. DMEM/F12 was then added to terminate digestion, and the tissue was pipetted several times to collect the supernatant and pass it through a 100 μm cell strainer. Repeat the above steps 2 $\sim$ 3 times to collect more bile duct cell clusters or liver progenitor cell precipitates. Matrigel was added according to the volume of the final cell pellet, evenly resuspended, and then 50 µL per well was added in a 24-well plate and incubated at 37℃ in an incubator for 15 min. After the Matrigel solidified, 500 µL of mouse liver expansion medium (EM) which contained various small molecule growth factors and amino acids was added to each well and cultured at 37 ℃ in an incubator. After 3 days, the number of cells rapidly increased, and individual organoids expanded into the spherical structure with a cavity. On day 6, the differentiation medium (DM) was added to promote the differentiation of organoids towards liver cell characteristics. It is worth noting that dexamethasone was added to the DM only in the last 3 days.

### Synthesis of GNS and GNB

According to the published method, GNSs and GNB were synthesized through the seed-mediated method [31]. Briefly, a HAuCl_4_ solution (0.01 M, 250 µL) was added into a CTAB solution (0.1 M, 9.75 mL), and then freshly prepared NaBH_4_ solution (0.01 M, 600 µL) was added. After mixing and stirring for 3 h, gold seed solution A was obtained. Next, a growth solution was prepared, consisting of H_2_O (190 mL), HAuCl_4_ (0.01 M, 4 mL), CTAB (0.1 M, 9.75 mL), and AA (0.1 M, 15 mL) solutions. The solution was quickly mixed until it became transparent, and then 180 µL of seed solution A was added rapidly and mixed thoroughly. The mixture was shaken evenly and kept overnight at 32 °C. to get gold seed solution B. Next, CTAC (0.025 M, 300 mL), HAuCl_4_ (0.01 M, 15 mL), AA (0.1 M, 7.5 mL), and solution B (OD = 3, 12 mL) were mixed thoroughly. After overnight incubation at 32 °C, the mixture was centrifuged at 7000 rpm for 6 min. The precipitate was collected and redispersed in CTAB solution (0.02 M, 300 mL), followed by 10 min of ultrasound treatment. HAuCl_4_ (0.01 M, 3 mL) was added and the mixture was placed at 60 °C for 2 $\sim$ 3 h, with shaking every 30 min. This process yielded the final GNSs.

The synthesis of GNBs is based on the following steps: A HAuCl_4_ solution (0.01 M, 250 µL) was added into a TX solution (0.15 M, 10 mL), and then the freshly prepared NaBH_4_ solution (0.01 M, 600 µL) was added under continuously stirring for 10 min to get the gold nanoseed solution. Next, a growth solution is prepared, which consists of TX (0.15 M, 400 mL), HAuCl_4_ (0.01 M, 20 mL), AgNO_3_ (10 mM, 2 mL), and AA (0.1 M, 6 mL) solutions. Once the mixture becomes transparent, the seeds (400 µL) are quickly added and shaken continuously for 5 min. The solution is then stored in a refrigerator at 4 °C.

### Characterizations

The images of GNSs/GNBs were obtained by TEM (JEM-2100, Japan). Specifically, we centrifuged the GNSs/GNBs at 6000 rpm twice and redispersed them in deionized water, then we carefully added the GNSs/GNBs solutions with appropriate concentrations dropwise onto a copper mesh for TEM characterizations. The size distribution was measured from the TEM images by ImageJ software. The dynamic light scattering (DLS) and average ζ potential were measured by a Zetasizer analyzer (Zetasizer-Nano ZS, UK). The ultraviolet-visible (UV-Vis) spectrum of GNSs/GNBs was obtained by a spectrophotometer (China). Flow cytometry was performed by a NovoCyte flow cytometer (Agilent, USA). Inductively coupled plasma mass spectrometry (ICP-MS) was carried out by an Optima 2100 instrument from PerkinElmer (Waltham, USA). Confocal laser scanning microscopy (CLSM) images were captured using a Nikon A1 camera (Tokyo, Japan). The RT-qPCR data was detected using the CFX384 Multiplate RT Fluorescence Quantitative PCR instrument (Bio-Rad, USA).

### Finite-difference time-domain (FDTD) simulations

The simulation was performed using Ansys Lumerical’s FDTD Solutions 2019b R6. In the simulation, an electromagnetic plane wave in the wavelength range from 400 to 1100 nm was launched into a box containing a target GNS model, and an electromagnetic plane wave in the wavelength range from 400 to 1800 nm was launched into a box containing a target GNB model. The mesh size was set at 0.5 nm in calculating the electric field enhancement contours, the absorption, scattering, and extinction spectra of the nanocrystals. The polarization direction of the incident light is set parallel to the length axis, exciting the longitudinal dipole plasmon resonance of the GNPs. The refractive index of the surrounding medium was set to be equal to that of water (1.33). The dielectric function of Au was acquired through fitting the measured data of Christy and Johnson. The GNS is modeled as a regular sphere with a diameter of 77 nm. The GNB model is composed of a central sphere connected to 6 approximately cylindrical branches, with a sphere diameter of 10 nm, branch tip diameter of 8 nm, branch base diameter of 12 nm, and branch length of 38 nm.

### Cellular uptake of GNPs

GNSs/GNBs was mixed with mercapto-modified Rhodamine B at room temperature for 4 h, avoiding light. Then, it was centrifuged and washed 3 times to ensure the removal of excess Rhodamine B. After incubating HepG2 cells or organoids with GNSs/GNBs of 10 µg/mL for different time, the cells or organoids were washed 3 times with PBS. The cells and organoids were fixed with 4% paraformaldehyde and stained with DAPI for 10 min. After washing 3 times with PBS, the cellular uptake was observed by CLSM imaging.

GNSs and GNBs were incubated with organoids for different time (6 h, 12 h, 24 h) at a concentration of 10 µg/mL. Then, the organoids were collected by centrifugation at 1200 rpm for 3 min. After digestion with aqua regia, the concentrations of Au were measured by ICP-MS (Agilent7700, USA).

### Cell and organoid viability assay

HepG2 cells were previously seeded in 96-well plates and adhered overnight. The cells were co-cultured with different concentrations of GNSs/GNBs (0, 10, 50, 100, 200 µg/mL) in DMEM for 24 h. Then, 10 µL of CCK8 detection solution was added to each well, and the cells were further incubated at 37 °C for 1 h. Afterward, the results were measured using a multi-functional microplate reader (Agilent, USA) at a wavelength of 450 nm.

After diluting Matrigel with DMEM/F12 at a ratio of 5–10 times, it was pre-coated on a 96-well plate with 40 µL per well. Then the plate was placed at 37 °C for 10 min. Subsequently, the organoids were added to the 96-well plate at a density of 100 per well, centrifuge the plate at 1000 rpm for 2 min. Organoids were incubated for 24 h with different concentrations of GNSs/GNBs (0, 10, 50, 100, 200 µg/mL) in DM. After discarding the old medium, 50 µL of detection solution was added to each well and shaken for 20 min. The chemiluminescent results were then measured using a multi-functional microplate reader.

### Impairment indicator detection

The levels of aspartate transaminase (AST), and alanine aminotransferase (ALT) in the supernatant gathered after the treatment with GNSs and GNBs at a concentration of 10 µg/mL were tested using a reagent kit (Nanjing Jiancheng Bioengineering Institute, Nanjing, China).

### Biological electron microscope

The organoids were collected and fixed in a 2.5% glutaraldehyde fixative solution. They were then dehydrated with different gradient solutions of ethanol and acetone, followed by embedding in resin and preparation of 70 $\sim$ 90 nm thin sections using an ultramicrotome. The sections were stained with lead citrate solution and 50% ethanol-saturated solution of uranyl acetate for 5 $\sim$ 10 min each, and then air-dried for observation of the ultrastructure of the organoid samples.

### Intracellular micro-signal detection

Detection of F-actin: After incubating 10 µg/mL of GNSs/GNBs with cells/organoids for 6 h, 4% paraformaldehyde was used to fix the cells/organoids. Rhodamine Phalloidin was diluted 1:500 in PBS and added to the samples to cover the volume of cells/organoids. The samples were then incubated at 37 ℃ for 30 min and washed 3 times with PBS. DAPI was used to stain the cell nuclei and incubated at room temperature for 10 min. After washing with PBS, CLSM images were obtained at 405 and 565 nm.

ROS Detection: After incubating 10 µg/mL of GNSs/GNBs with cells/organoids for 6 h, the DCFH-DA probe was diluted 1:1000 in serum-free medium and added to cover the volume of cells/organoids. The samples were then incubated at 37 ℃ for 20 min and washed 3 times with serum-free medium. CLSM images were obtained at 488 nm.

Detection of mitochondrial membrane potential (MMP): The JC-1 Mitochondrial Membrane Potential Detection Kit was used according to the instructions to detect changes in mitochondrial membrane potential in cells and organoids under different stimuli, serving as evidence for early apoptosis detection. CLSM images were obtained at 514 and 590 nm.

Detection of intracellular lipid droplets: After incubating 10 µg/mL of GNSs/GNBs with cells/organoids for 6 h, Nile Red was diluted to 1 µM in PBS and added to the cells/organoids. Then the samples were incubated at 37℃ for 30 min and washed with PBS. CLSM images were obtained at 550 nm. Semi-quantitative data was obtained from the total fluorescence intensity of CLSM images.

### HE staining

Preparation of sample embedding and slicing: After dehydration and paraffin embedding, the samples were subjected to alcohol dehydration at different concentrations. After embedding, they were cooled and shaped at -20 °C. The paraffin blocks were then placed in a paraffin microtome and cut into several 4 μm thick sections, which were spread onto glass slides and baked at 60 °C for 1 h. They were then stored at room temperature.

HE staining: After dehydration with different concentrations of alcohol, the paraffin sections were stained with hematoxylin-eosin (Harris) staining solution for 3–5 min, followed by a 2 min water rinse and differentiation with 0.8-1% hydrochloric acid alcohol for a few seconds, followed by another 1 min water rinse. Subsequently, the sections were immersed in eosin solution for 2 s, and then gradually dehydrated in 95% ethanol and absolute ethanol for 2 min. Finally, the sections were mounted with neutral gum for observation of the HE staining results under a microscope.

### Immunofluorescence staining

After slicing the paraffin, the slices are dehydrated sequentially with xylene and ethanol. After blocking with 10% serum for 30 min, the primary antibody is added and incubated overnight at 4 °C in a refrigerator. The slices are washed 3 times with TBST and then each slice is added to the secondary antibody working solution. They are then incubated at 37 °C for 45 min. After another 3 washes, the slices are incubated with DAPI at room temperature for 5 min. After a thorough rinse, the slices are mounted and images are obtained using CLSM.

### Real-time qPCR analysis

After incubating organoids with 10 µg/mL GNSs/GNBs for 12 h, the total RNA of the organoids was extracted using the TRIzol Plus RNA Purification Kit (Thermo Fisher, USA). The extracted RNA was then reverse-transcribed into cDNA using the 1st-Strand cDNA Synthesis Kit (Qiagen, Germany). The cDNA was fluorescently labeled with SYBR Green (Thermo Fisher, USA) and detected using the CFX384 Real-Time PCR System (Bio-Rad, USA). Each sample was repeated three times, and the relative expression levels of each gene were statistically analyzed using formula 2^(Ct reference gene − Ct target gene)^. The primer sequences and reaction conditions used are shown in the following table. 1.

**Table 1:** Real-Time PCR Primers and Conditions

Gene	Genbank Accession	Primer Sequences(5’to3’)	Size(bp)	Annealing (℃)
Mouse GAPDH	GU214026.1	GAAGGTCGGTGTGAACGGATTTG	127	60
Mouse AFP	NM_007423.4	GCTCACATCCACGAGGAGTGTT	153	60
		GCAGAAGCCTAGTTGGATCATGG		
Mouse ALB	NM_009654.4	CAGTGTTGTGCAGAGGCTGACA	107	60
		CTGGAGCACTTCATTCTCTGACG		
Mouse CFTR	NM_021050.2	CTTCGCTGGTTGCACAGTCA	105	60
		GAGTCGTACTGCCAGACATTG		
Mouse CYP3A11	NM_007818.3	CAGCACTGGTCAGAGCCTGAA	132	60
		GAGAGCAAACCTCATGCCAAGG		
Mouse EPCAM	NM_008532.2	GTCATTGTGGTGGTGTCATTAGC	106	60
		CCATCTCCTTTATCTCAGCCTTCT		
Mouse HNF4A	NM_008261.3	GCGAACTCCTTCTGGATGACC	104	60
		CAGCACGTCCTTAAACACCATGG		
Mouse Krt19	NM_008471.3	GGCGAGCTGGAGGTGAAGA	132	60
		GGAGTTGTCAATGGTGGCACCAA		
Mouse SOX9	NM_011448.4	ACCCACCACTCCCAAAACC	114	60
		ATGTCCACGTCGCGGAAGT		

### Animals and experimental design

All animal procedures were performed by the Guidelines for Care and Use of Laboratory Animals of Zhejiang Sci-Tech University and approved by the Animal Ethics Committee of Zhejiang Sci-Tech University. Male C57BL/6 mice (6 weeks, 16 ± 1 g) were fed in a ventilated environment with a humidity of 50%. The cages are cleaned in advance and sterilized with UV light. Clean food and water are provided, and the lighting is switched between day and night every 12 h.

The mice were given 2–3 days to adapt to the environment and were then randomly divided into 3 groups (with 4 mice for each group, *n* = 4). They were injected with sterile PBS and PBS solutions containing GNSs and GNBs. The NPs were intravenously injected at a dosage of 10 mg/kg. On the 7th day, the mice were euthanized, and blood serum was collected for analysis of blood biochemistry indicators. Additionally, organs and tissue slices were stained with HE for histological analyses.

### Serum biochemical index

To evaluate the liver function of mice, blood samples were centrifuged at 3000 rpm for 10 min at 4 °C to collect plasma. ALT, AST, albumin (ALB) and alkaline phosphatase (ALP) levels were measured using an automatic chemistry analyzer (Celltac, MEK-6358; Nihon Kohden Co., Tokyo, Japan) to evaluate the liver functions.

### Statistical analysis

All quantitative results are presented as mean ± standard deviation (SD). Non-paired t-tests were used for statistical analysis of comparisons between two groups, and one-way analysis of variance (ANOVA) was used for comparisons among multiple groups. Data analysis was performed using GraphPad Prism software (GraphPad Software, USA). Significance was defined as ***P < 0.001, ** *P* < 0.01, or *P < 0.05.

## Results and discussion

### Generation of mice Hep-orgs

**Fig. 1:**
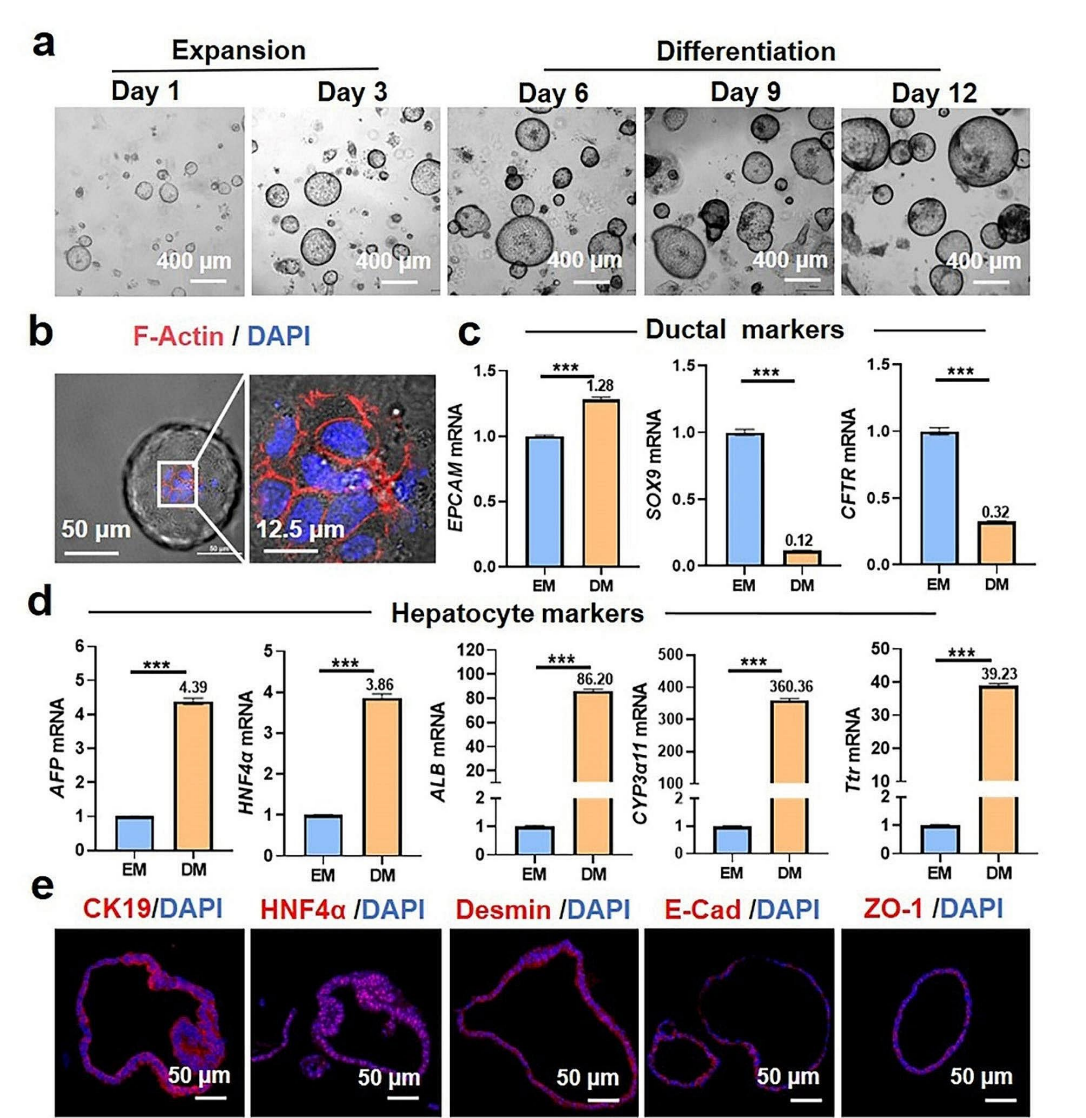
Generation of mice Hep-orgs with hepatocyte characteristics. (**a**) Bright-field optical images of organoids in EM and DM. (**b**) CLSM images of Hep-orgs stained with F-actin and DAPI, showing a single plane in a 3D scan. (**c**) Relative gene expressions of EPCAM and ductal markers (Sox9, CFTR) in organoids cultured in EM and DM, with GAPDH as the internal control. (**d**) Relative gene expressions of hepatocyte markers (AFP, HNF4α, ALB, CYP3α11, Ttr) in organoids cultured in EM and DM, with GAPDH as the internal control. (**e**) CLSM images showing the expressions of liver markers CK19, HNF4α, Desmin, E-Cad, and ZO-1 in Hep-orgs. All data are presented as mean ± SD (*n* = 3). *P < 0.05, **P < 0.01, ***P < 0.001

The basic unit of the liver is primarily composed of two types of epithelial cells (hepatocytes and bile duct epithelial cells) and several non-epithelial cells (stellate cells, kupffer cells, endothelial cells, and matrix cells) [32]. A long-term expansion culture system derived from adult liver stem cells of mice has been developed to generate Hep-orgs. Single liver progenitor cells have the potential to differentiate into bile duct-like organoids with bile duct markers or Hep-orgs with hepatocyte markers [29]. Representative images of the growth of Hep-orgs are shown in Fig. 1a. The organoids in EM continued to expand like inflating balloons. When the diameter of the organoids stopped increasing, their surface became wrinkled, as if deflated and collapsed (Additional file 1: Fig. S1a). At this point, passaging was performed to obtain more liver organoids. The size change of organoids in EM during its growth process can be observed (Additional file 1: Fig. S1b). The HE staining and immunohistochemistry (IHC) images demonstrated its morphological structure and expression of zona occludens-1 (ZO-1) (Additional file 1: Fig. S1c). Rhodamine-phalloidin was used to label F-actin. Since the organoids are enlarged hollow spheres, single-layer images from 3D scans were selected to observe the ordered arrangement of multiple cells, with F-actin closely connected to support the large sphere (Fig. 1b). RT-qPCR was used to detect the relative mRNA expression levels of EPCAM, SOX9, and CFTR, which are specific genes of bile duct structures. The results showed that there were more bile duct markers in EM organoids than those in DM organoids. (Fig. 1c). Then, classic hepatocyte markers of alpha-fetoprotein (AFP), hepatocyte nuclear factor 4 alpha (HNF4α), albumin (ALB), cytochrome P450 (CYP3α11), and transthyretin (Ttr) were detected both in the DM and EM organoids. The levels of hepatocyte markers in DM organoids are significantly higher than those in EM organoids, indicating the potential of DM organoids to differentiate into liver cells (Fig. 1d). The characteristics of the Hep-orgs were confirmed by immunofluorescence staining. Cytokeratin 19 (CK19), HNF4α, desmin, E-cadherin (E-cad), and ZO-1 were expressed at specific locations and with different degrees of the organoids (Fig. 1e). In addition, we achieved long-term in vitro, cryopreservation, and recovery of the Hep-orgs, which showed no significant differences compared to the continuously cultured organoids (Additional file 1: Fig. S1d). Thus, we have successfully constructed Hep-orgs with liver characteristics through in vitro growth for further material evaluation applications.

### Physicochemical characteristics of GNSs/GNBs

**Fig. 2:**
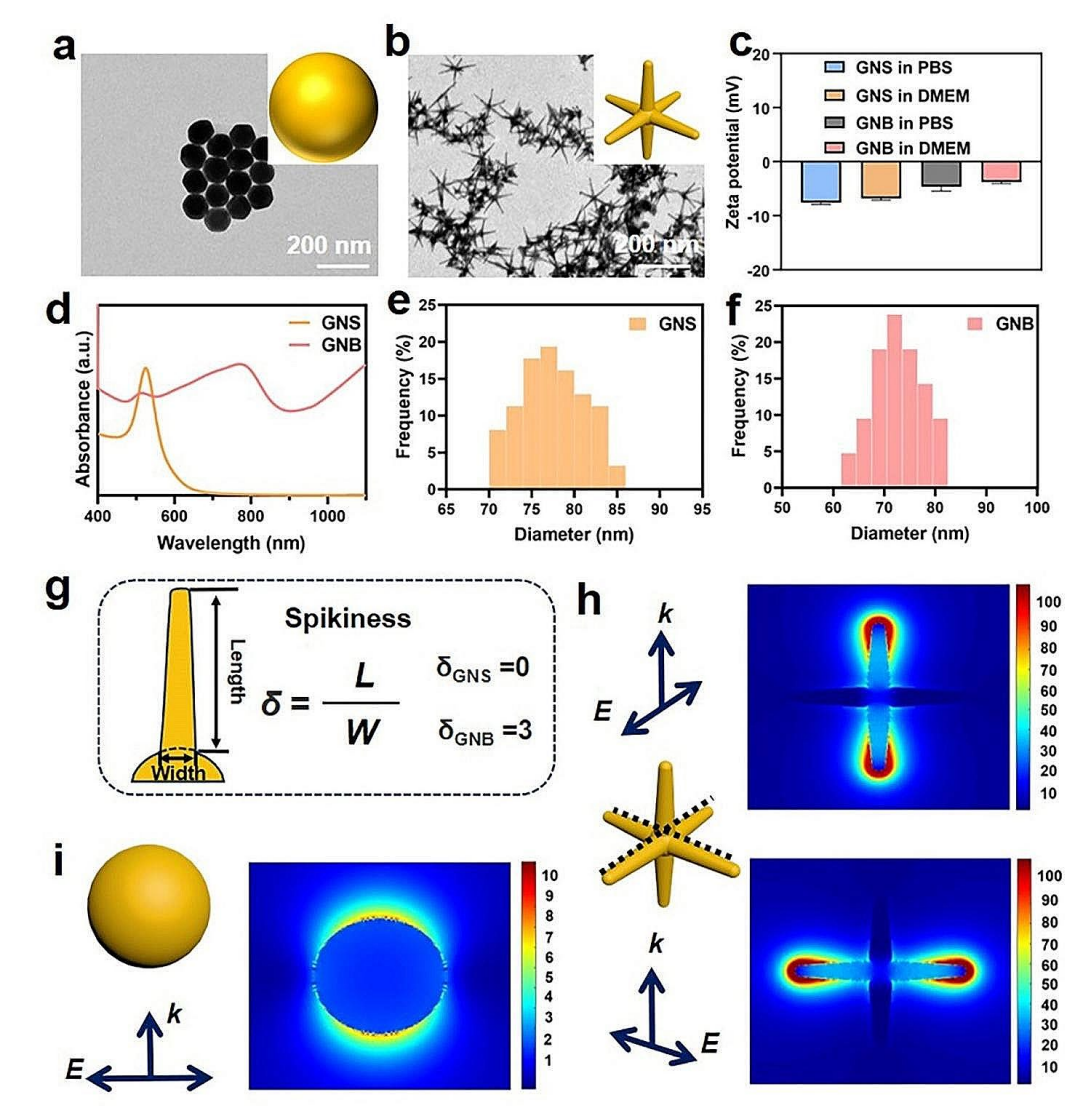
Characterizations of GNSs and GNBs. (a) TEM image of the GNSs, with a schematic representation in the upper right corner. (**b**) TEM image of GNBs with a schematic representation in the upper right corner. (**c**) Zeta potential graphs show the even dispersion of GNSs and GNBs in different solutions (PBS and DMEM). (**d**) Extinction spectra of GNSs and GNBs. (**e**) Particle size distribution of GNSs. (**f**) Particle size distribution of GNBs. **(g)** The spikiness of GNSs (δ = 0) and GNBs (δ = 3). (**h,i**) FDTD simulations of the GNS (**h**) and GNB (**i**), electric field enhancement contours simulated at the plasmon resonance wavelengths under the axial/longitudinal and transverse excitation polarization directions, respectively

We prepared GNSs using a seed-mediated method according to a previous report, which involves the overgrowth of small-sized GNSs and subsequent mild oxidation [31]. The synthesis of GNBs refers to a publicly available method [33]. The TEM image shows that GNSs have an approximately spherical shape and a good monodispersity (Fig. 2a), while GNBs have a spike-branched shape derived from a central point, with an average number of branches of 6 (Fig. 2b). Both GNSs and GNBs exhibited negative potentials even in different media. The average potential of GNSs was − 7.6 mV in PBS and − 6.83 mV in DMEM, while the average potential of GNBs was − 4.68 mV in PBS and − 3.8 mV in DMEM. Similar potentials can rule out the influence of different surface charges on cellular internalization differences (Fig. 2c). The branched shape of GNBs is highly anisotropic, and the positions of its absorption peaks are related to the migration of gold atoms from the high-energy site at the tip to the low-energy site at the core [34]. The results show that GNSs have an extinction peak at 520 nm, and GNBs have two plasmon resonance peaks at 520 and 770 nm, indicating that there are two different plasmon modes for GNBs (Fig. 2d).

To control the size of the GNPs within an approximate range and eliminate the potential impact of size differences on cytotoxicity. Both two types of GNPs have a similar size and a narrow size distribution, with an average diameter of 77 ± 4 nm for GNSs and 72 ± 4 nm for GNBs (Fig. 2e,f). The DLS results showed that the average size of GNSs was 87.36 nm and the average size of GNBs was 88.81 nm, which were larger than those measured from TEM images owing to the interaction of the surrounding water molecules (Additional file 2: Fig. S2a). In order to accurately study the relationship between the toxicity and the spike structure, we have calculated and quantified the tip structure in the form of spikiness and curvature. Spikiness is the ratio between branch length and bottom width of GNBs, which can separate spiky morphology cues from complex physiochemical ones [12]. According to Fig. S2b, the spikiness of GNSs is 0 and that of GNBs is 3. The tip radius of GNBs is 4 nm. The curvature ratio between GNB and GNS is 9.5:1 (Fig. 2g, and Additional file 2: Fig. S2b). FDTD simulations were performed to visualize the tip effect of the GNB (Fig. 2h,i and Additional file 3: Fig. S3). The simulated GNS and GNB structures were modeled according to the measured geometrical parameters. Electric field enhancement contours indicated that the largest electric field intensity was located at the sharp tip region of the GNB structure, demonstrating the tip-induced electric field enhancement. It is precisely this type of nano spike that may easily cause membrane damage, causing imbalanced osmotic pressure inside and outside the membrane, thereby increasing cytotoxicity [35].

### GNSs/GNBs internalized by cells and hep-orgs

Due to the essential role that Matrigel plays during the culture of organoids, many researchers thought that Matrigel could hinder the entry of materials into organoids, thereby affecting the true evaluation of materials by organoids [36]. In the study of the interaction between organoids and NPs, the size of organoids may affect the exposure and distributions of NPs, and larger organoids are more likely to accumulate NPs [37]. In addition, the size of organoids has an impact on the transport and movement of NPs. Owing to the structural heterogeneity of organoids from different tissue sources, the size of organoids is crucial for predicting the behavior of NPs. It is worth mentioning that organoids of different sizes may lead to reduced reproducibility of experimental results. At present, there is no relevant standard to specify the size of organoids for evaluating NPs, the “unified” treatment of organoids is still a great challenge [38]. Therefore, in this study, we used the same batch of Hep-orgs with the same culture time and conditions to reduce the objective influence of the organoid model itself on the internalization and operation of NPs.

**Fig. 3:**
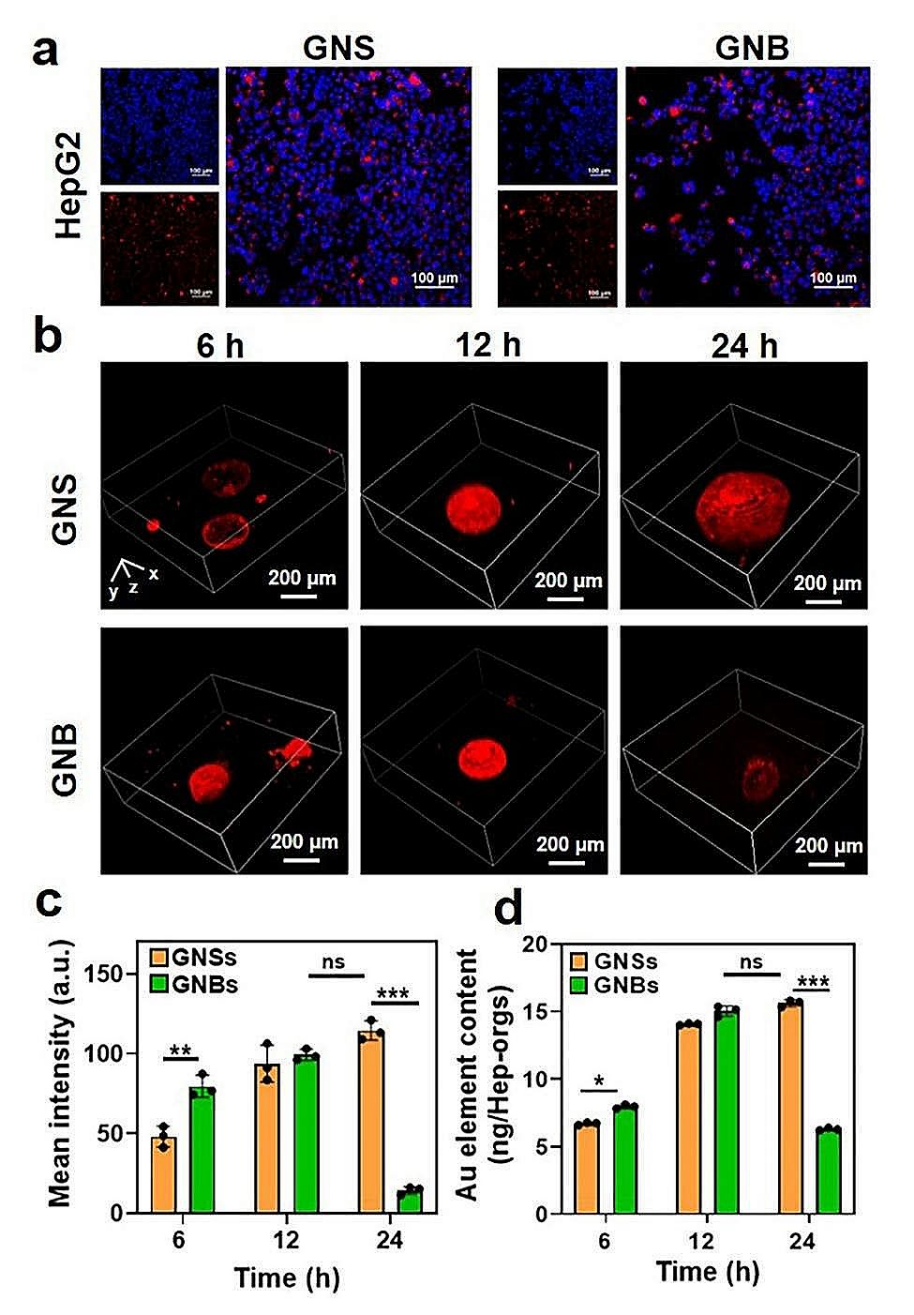
Internalization of GNSs and GNBs by HepG2 cells and Hep-orgs. (**a**) CLSM images showing the internalization of GNSs and GNBs by HepG2 cells. (**b**) CLSM images showing the internalization of GNSs and GNBs by Hep-orgs at different time periods (6 h, 12 h, and 24 h). Red: GNSs/GNBs labeled with Rhodamine B. The scanning height was 200 μm with a scale interval of 10 μm. (**c**) Mean fluorescence intensity of the internalization of GNSs and GNBs by Hep-orgs at different time periods (6 h, 12 h, 24 h). (**d**) Au element contents after the incubation with GNSs and GNBs at the concentration of 10 µg/mL for different time periods (6 h, 12 h, 24 h)

To investigate whether GNSs/GNBs have been internalized by HepG2 cells or Hep-orgs, we labeled GNSs/GNBs with mercapto-modified rhodamine B to form stable Au-S covalent bonds and then co-cultured them with HepG2 cells and Hep-orgs for 6 h. The distributions of both types of GNPs were observed under CLSM. In HepG2 cells, GNSs/GNBs were found to aggregate around the cell nucleus, indicating the successful internalization by the cells (Fig. 3a). Internalization of GNSs and GNBs was observed in Hep-orgs, with the degree of internalization correlating with the duration of incubation (Fig. 3b). More GNBs were taken up by the Hep-orgs compared to GNSs at 6 h, reflecting the fact that GNBs with nanospikes displayed easy penetration into the organoids. At 12 h, GNSs and GNBs exhibited similar levels of fluorescence, and the accumulation and aggregation of GNSs and GNBs were observed in the cavities of Hep-orgs (Additional file 4: Fig. S4). The unique 3D structure of organoids may accumulate more nanoparticles than a monolayer cells layer with the same number of cells. At 24 h, the fluorescence of GNBs was significantly reduced, which can be attributed to the disintegration of the Hep-orgs to ruptured cells and then the release of internalized GNBs. The above results were further confirmed by the mean fluorescence intensity of the CLSM images (Fig. 3c) and the Au element contents tested by ICP-MS (Fig. 3d). It has been shown that the internal content of NPs is one of the key factors affecting the interaction of NPs with organelles upon the exposure [39]. Therefore, the evaluation of the internalization of GNSs and GNBs in Hep-orgs by fluorescence intensity and ICP-MS confirmed that the toxicity of GNBs was shape-dependent rather than dose-dependent.

#### Biological toxicity of GNSs/GNBs in cells and Hep-orgs

**Fig. 4:**
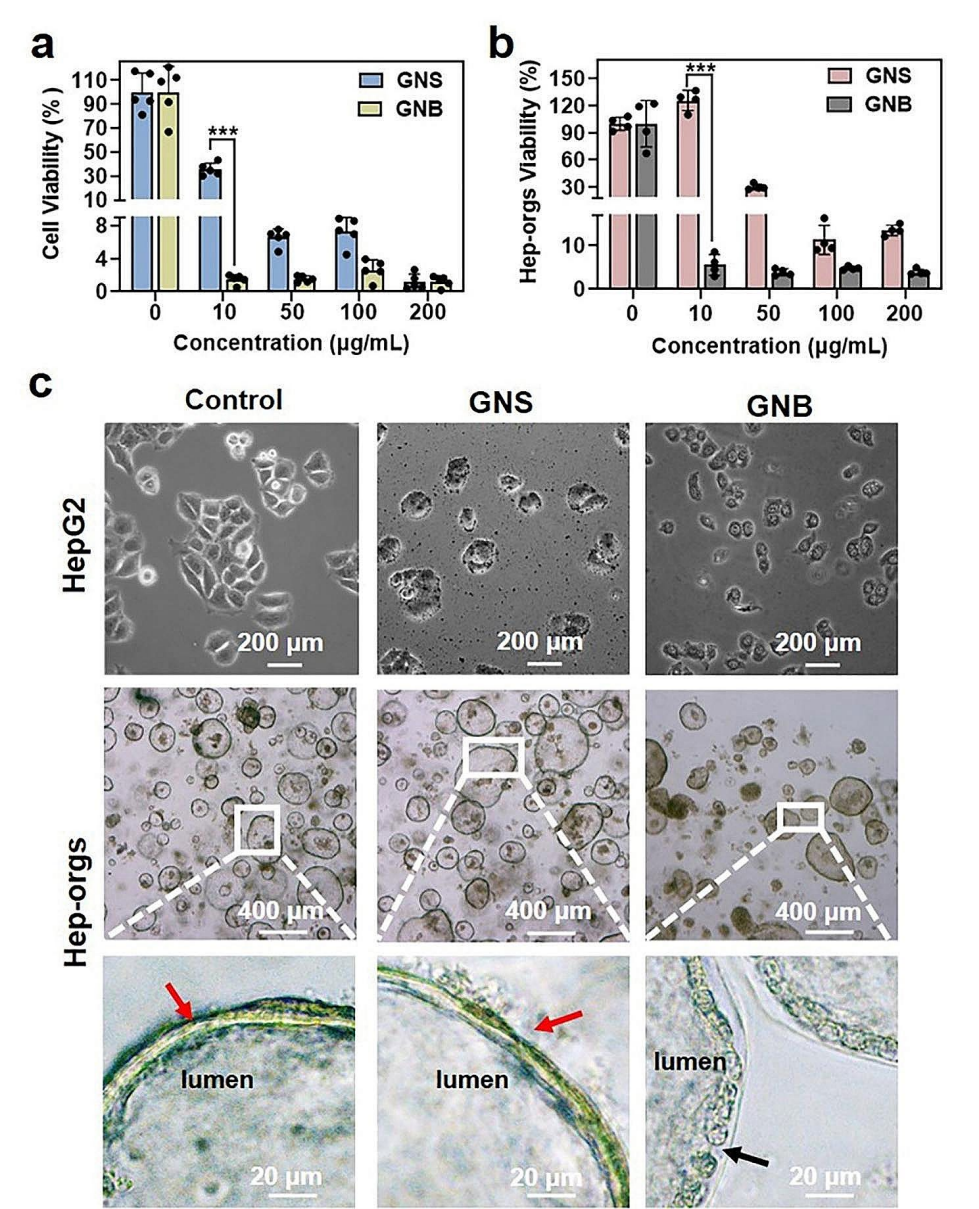
The effects of GNSs/GNBs on the cytotoxicity and morphology of HepG2 cells and Hep-orgs. (**a**) Cytotoxicity of GNSs and GNBs on HepG2 cells. (**b**) The results of GNSs and GNBs on ATP production in Hep-orgs. (**c**) Bright-field optical images of HepG2 cells and Hep-orgs after treatment with GNSs and GNBs. Red arrows point to the outer wall of the Hep-orgs, and black arrow points to the enlarged intercellular space on the outer wall of the Hep-orgs. All data are presented as mean ± SD (the black dots represent the number of samples). *P < 0.05, **P < 0.01, ***P < 0.001

HepG2 cells and Hep-orgs were used to screen the biological toxicity of GNSs and GNBs. GNSs and GNBs with different concentrations were co-cultured with cells and Hep-orgs for 24 h. The viability of the cells was measured using CCK-8 assay and the growth vitality of the Hep-orgs was quantified by measuring the ATP content. We found inconsistent toxicity trends for GNSs and GNBs. At the cellular level, both GNSs and GNBs exhibited toxicity. GNSs showed dose-dependent toxicity, while GNBs showed greater cellular toxicity at all doses (Fig. 4a). This difference was amplified in the organoids, where GNSs did not display toxicity at a low concentration of 10 ug mL^–1^ but the toxicity of GNBs remained. We found that the IC50 in Hep-orgs was much higher than that in the cell model. This difference suggests that organoids, as a 3D model, have greater resistance to adverse conditions (Fig. 4b).

Morphologically, HepG2 cells were significantly disturbed by GNSs/GNBs. Tight intercellular connections were disrupted and the orderly spindle-shaped morphology was transformed into disordered shapes, indicating the generation of cell toxicity. In Hep-orgs, no obvious effect on individual organoid morphology was observed with GNSs treatment. However, the spherical morphology of Hep-orgs was significantly different after GNBs treatment, and the color of organoids became darker (Fig. 4c). The outer wall of the organoids is an important structural feature, as its integrity allows organoids to function as independent structural units, forming a barrier between organoids and the matrix to prevent leakage of contents [40]. Therefore, we further observe the outer wall structure of the randomly selected Hep-orgs. It was found that the outer wall did not show significant morphological changes in the control and GNSs treatment groups (red arrows). However, in the Hep-orgs group treated with GNBs, the cells composing the outer wall were no longer tightly connected as a cohesive structure. Instead, they appeared as a scattered ring of cells with gaps in between (black arrows). This confirms that exposure to GNBs with spikiness value of 3 can cause loss of intercellular junction integrity in liver cells and damage to cell membranes [41]. We speculate that the nano spikes of GNBs make them easier to penetrate the cell membrane, cause physical damage and alter the integrity of the cell membrane. This process is visualized in vitro based on Hep-orgs. Therefore, we believe that the Hep-orgs, which contain multiple cell types and tight intercellular connections, allow for more efficient intracellular and extracellular signal communications and provide a three-dimensional perspective that is closer to the in vivo physiological environment. Thus, it is a more accurate in vitro model for evaluating the toxicity of NPs compared to individual cells.

### Identification of the cytoskeleton damage, generated ROS, and mitochondria damage in Hep-orgs

**Fig. 5:**
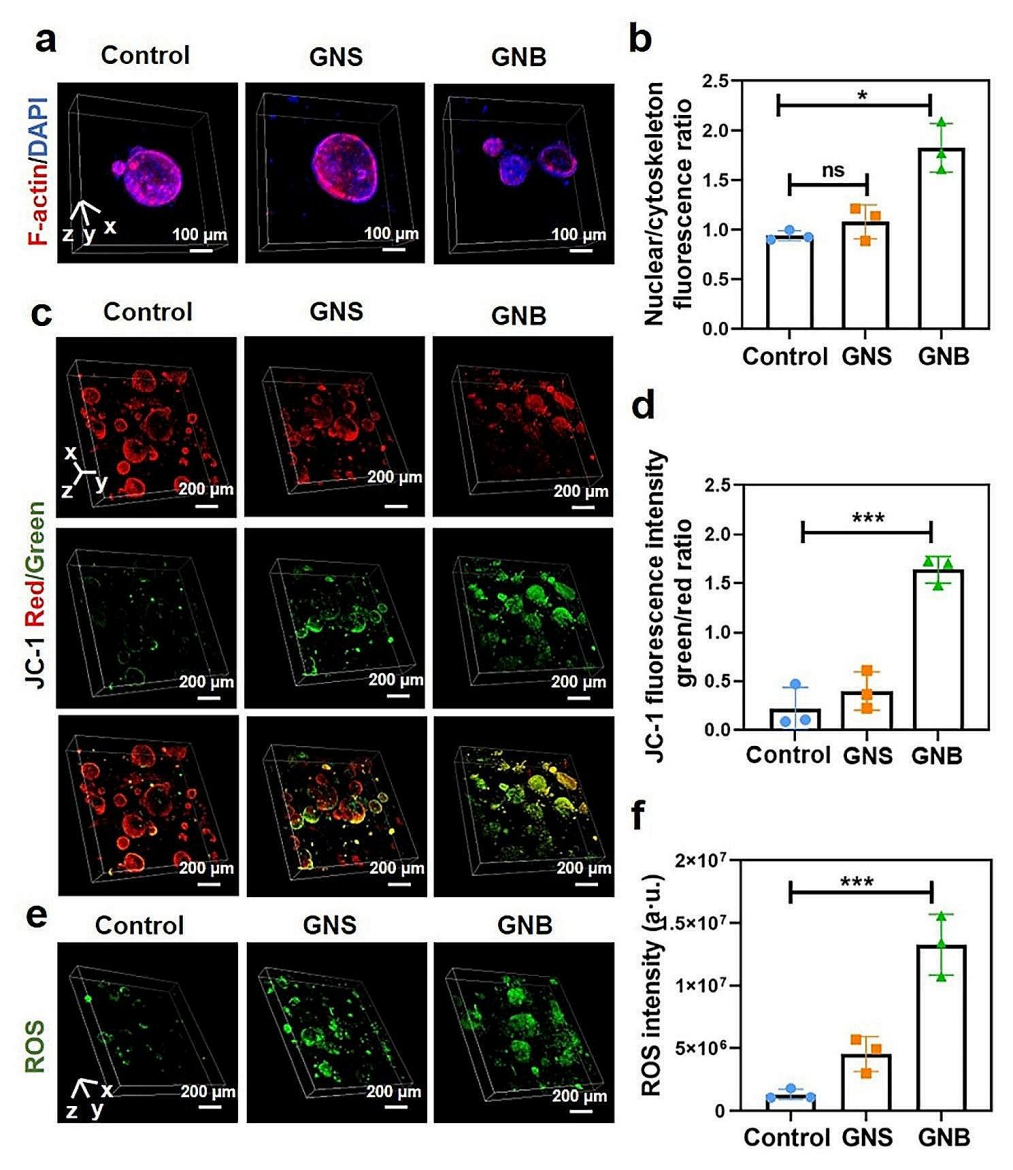
The effects of GNSs/GNBs on the structural and physiological states of Hep-orgs. (**a**) CLSM 3D composite images of Hep-orgs cell cytoskeleton after treatment with GNSs and GNBs. The scanning height was 200 μm with a scale interval of 5 μm. (Red: F-actin; Blue: DAPI) (**b**) Corresponding fluorescence intensity ratio of nuclear/F-actin in Hep-orgs. (**c)** CLSM 3D composite images of MMP changes in Hep-orgs after the treatment with GNSs and GNBs. The scanning height was 300 μm with a scale interval of 5 μm. (Red: J-aggregates; Green: Monomer) (**d**) Ratio of JC-1 green/red fluorescence intensity in Hep-orgs. (**e)** CLSM 3D composite images of ROS production in Hep-orgs after the treatment with GNSs and GNBs. The scanning height was 250 μm with a scale interval of 5 μm. (**f**) Corresponding fluorescence intensity of ROS production in Hep-orgs. All data are presented as mean ± SD (the dots represent the number of samples). *P < 0.05, **P < 0.01, ***P < 0.001

The main components of the cytoskeleton, which contributes to cellular support and intercellular connections, are microtubules, microfilaments, and intermediate filaments [42]. They play important roles in maintaining cell shape, contributing to cell division, and participating in cell movement and intracellular transport [43]. We stained F-actin to observe the cell structure changes in HepG2 and Hep-orgs, which are caused by microfilaments. Under the treatments with GNSs and GNBs, although the skeleton morphology of HepG2 cells was altered (Additional file 5: Fig. S5a), there was no significant differences in the statistics of fluorescence quantification (Additional file 5: Fig. S5b). It is worth mentioning that the 3D scans by CLSM help us better understand the assembly structure of F-actin in Hep-orgs. The retreat of cell skeletons was observed after treatment with GNSs and GNBs (Fig. 5a). The fluorescence statistical results of the corresponding nucleus/F-actin were also displayed in Fig. 5b, reflecting the higher sensitivity of the Hep-orgs and the more intuitive visualization of the results than those of 2D cells.

Furthermore, mitochondrial damage and oxidative stress induced by metal NPs are the main reasons for their cellular toxicity [44]. We examined the changes in MMP and the generation of ROS in HepG2 and Hep-orgs. Under GNSs treatment, the MMP of Hep-orgs decreased, indicating a disruption of mitochondrial membranes due to the acute stress from GNSs. When Hep-orgs were treated with GNBs, this change was greatly amplified. The alteration in the potential difference across the mitochondrial membrane would affect the electron transfer in the mitochondrial respiratory chain, leading to a decrease in ATP production in Hep-orgs (Fig. 5c). The ratio between green fluorescence and red fluorescence in MMP intuitively shows that GNBs caused extremely significant mitochondrial damage (Fig. 5d). However, MMP alterations did not show statistically significant differences in HepG2 cells (Additional file 5: Fig. S5c,d). Furthermore, a pronounced enhancement of ROS levels was observed in Hep-orgs treated with GNBs (Fig. 5e), and this change was also visually apparent in the fluorescence intensity of the same scanning area (Fig. 5f). Identically, this result was also observed in HepG2 cells (Additional file 5: Fig. S5e,f).

To further reveal the mechanism of hepatotoxicity induced by GNSs and GNBs, liver injury related indexes such as AST and ALT were also detected both in HepG2 cells and Hep-orgs. ALT is predominantly found in the cytoplasm of hepatocytes. AST is mainly distributed in hepatocyte mitochondria, with a small portion being distributed in the cytoplasm. As consistent with our assay of mitochondrial potential, the AST activity was significantly increased after the treatment with GNBs, and exhibited a higher level in Hep-orgs than HepG2 cells, which can be attributed to the enhanced hepatocyte function in the 3D microenvironment (Additional file 6: Fig. S6a). ALT viability increased after both GNSs and GNBs treatments, with it possessing a slightly higher level after the GNBs treatment (Additional file 6: Fig. S6b). The aforementioned results suggest that the hepatotoxicity of GNBs originates from their damage to mitochondria. More importantly, Hep-orgs have been proved that they can be used to reveal the possible mechanism of shape-induced hepatotoxicity with a higher sensitivity.

The differences caused by GNSs and GNBs can be attributed to their completely different shapes. The size of the nano-spikes at the tip of the GNBs was measured to be 8 ± 2 nm. It has been confirmed that the spiky nanostructure at the nanoscale can cause physical damage to cell membranes and organelles [45], and this physical damage can quickly affect the physiological function of Hep-orgs. In a brief summary, Hep-orgs exhibit a unique sensitivity compared to the cellular models, which can be attributed to the fact that organoids possess the ability to simulate the complex physiology in vivo. The special structure allows them for more frequent intercellular and cell-matrix signaling, which is superior to cell models in terms of reliability and visualization of results.

**Fig. 6:**
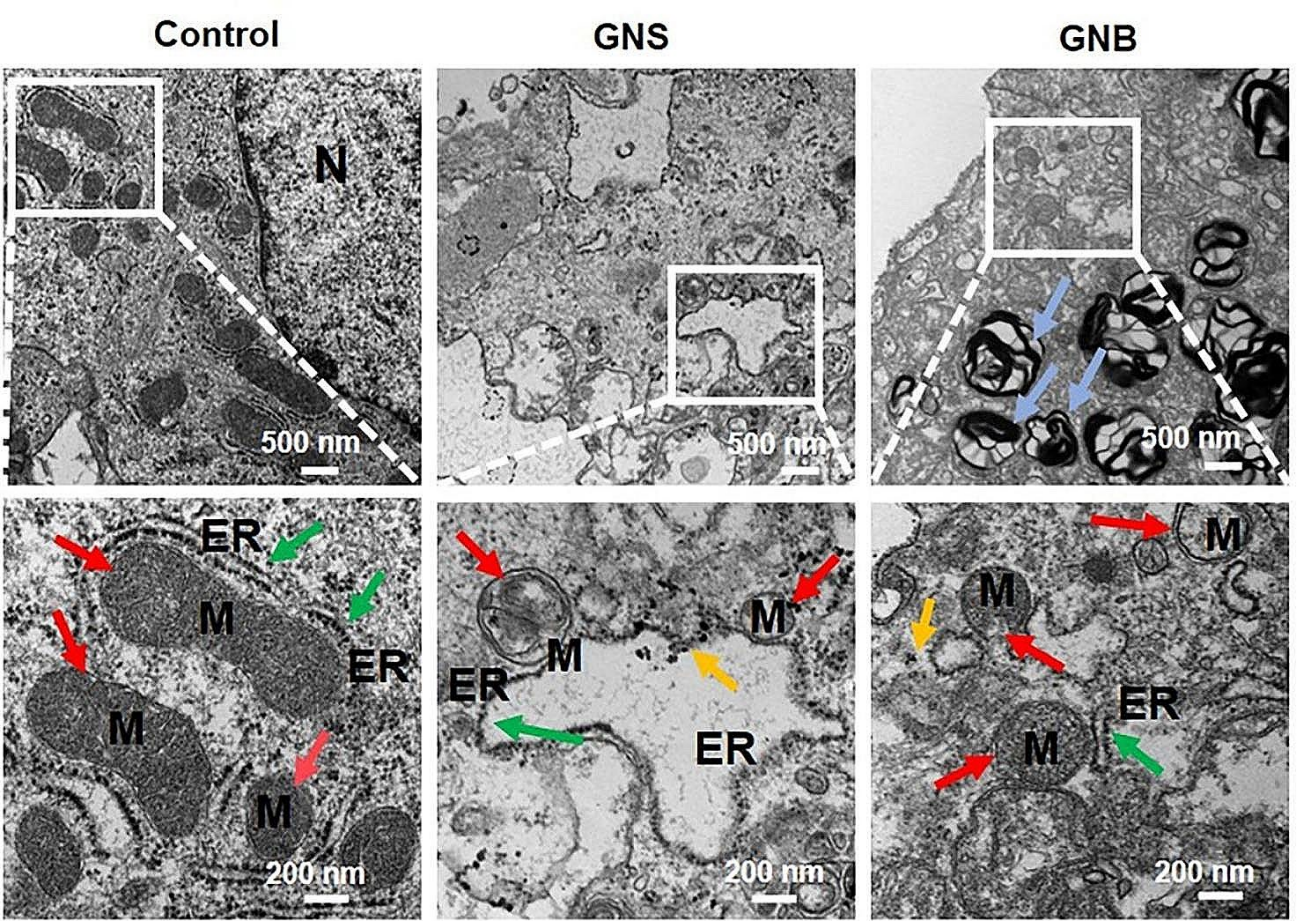
Representative TEM images of mitochondrial morphology and structure in Hep-orgs. Red arrows point to the mitochondria, green arrows point to the ER, yellow arrows point to the location of the internalized GNSs/GNBs, and blue arrows point to the autophagosome

To further investigate the GNPs’ impact on the organelles of Hep-orgs, we conducted TEM observations on Hep-orgs co-cultured with GNSs/GNBs (Fig. 6). The TEM images of the control group showed abundant mitochondria and endoplasmic reticulum (ER). The orderly arrangement of mitochondrial cristae is observed. The ER structure was clear, and multiple ribosomes could be observed. Under the influence of GNSs, the number of mitochondrial cristae and ribosomes decreases. Particularly, GNBs caused a rupture of the mitochondrial membrane and fragmentation of the endoplasmic reticulum membrane. It is worth noting that Hep-orgs produced autophagosome structures in response to the external stimuli of GNBs. These results suggest that acute exposure to GNBs can significantly cause mitochondrial damage and ER stress in Hep-orgs.

### Lipid accumulation in hep-orgs

**Fig. 7:**
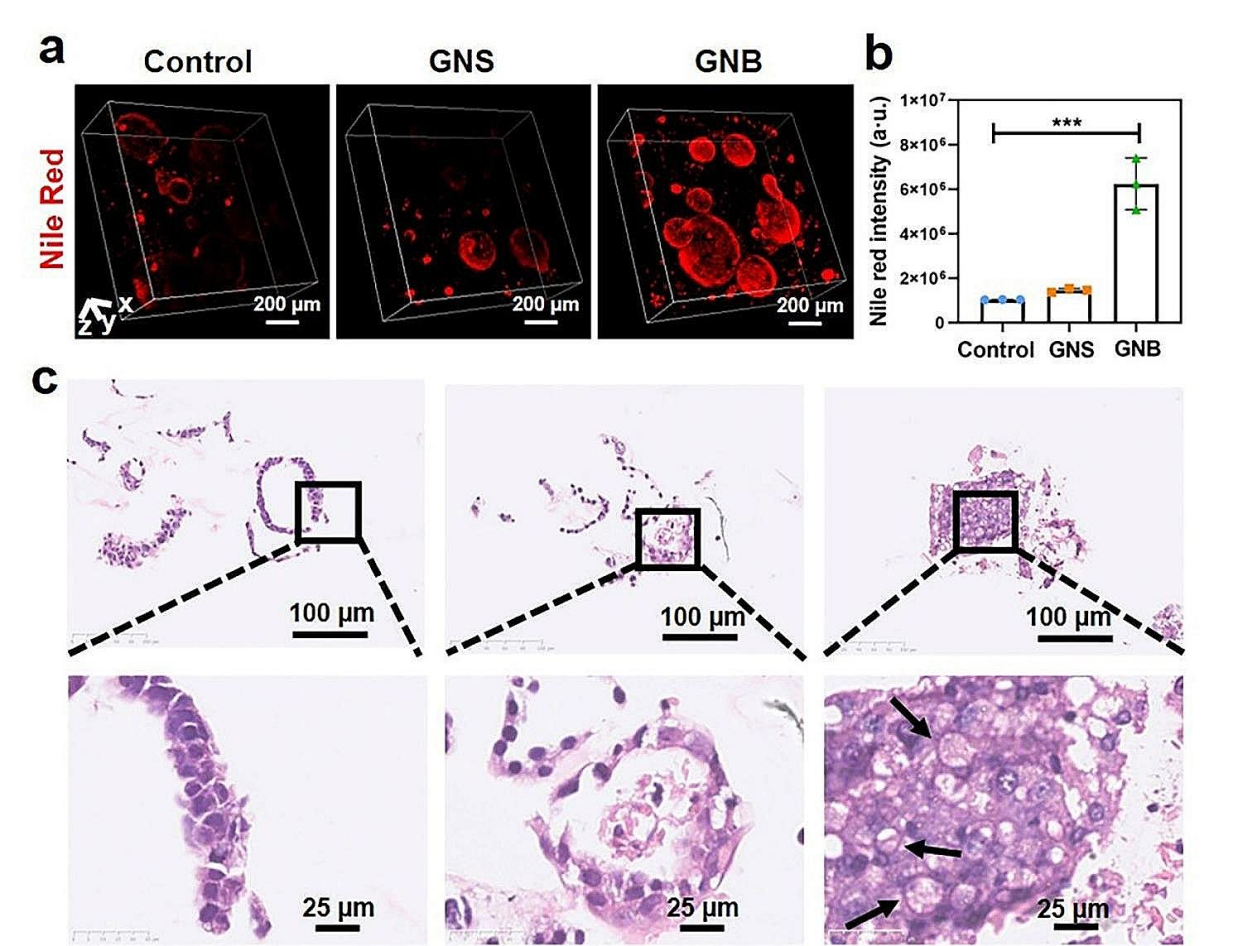
GNSs and GNBs caused metabolic abnormalities in Hep-orgs. (**a**) CLSM 3D composite images of internal lipid in Hep-orgs stained with Nile Red after treatment with GNSs and GNBs. (**b**) Corresponding fluorescence intensity plot. (**c**) Representative images of Hep-orgs stained with HE after treatment with GNSs and GNBs. Black arrows indicate the sites of lipid vacuolation. All data are presented as mean ± SD (the dots represent the number of samples). *P < 0.05, **P < 0.01, ***P < 0.001

The robust metabolic functions of the liver come from the thousands of mitochondria within each liver cell [46]. As the energy factory in cells, mitochondria maintain the normal physiological functions of cells through the metabolism of glucose, amino acids, and fatty acids [47]. Exposure to metal NPs may cause disruptions in the mitochondrial metabolism of fat. Previous research has reported that an increased lipid droplet deposition was observed in the liver cells of rats continuously injected with silver NPs [48]. Additionally, lipid degeneration was observed in rats after treatment with 10 nm gold nanoparticles [49]. Therefore, we investigated the effects of GNSs and GNBs on lipid metabolism in the Hep-orgs model through Nile Red staining and HE staining. The lipid accumulation in Hep-orgs induced by GNBs was significant (Fig. 7a). The fluorescence intensity from CLSM visually demonstrated this phenomenon (Fig. 7b). Pathological results of lipid degeneration caused by GNBs were also observed in HE staining (Fig. 7c), and abnormal lipid metabolism can also be observed in Hep-orgs. This result can be attributed to the spike structure of GNBs disrupting the structure of the mitochondrial membrane and endoplasmic reticulum membrane, blocking the metabolic pathway of lipids in cells.

### Liver damage in vivo

**Fig. 8:**
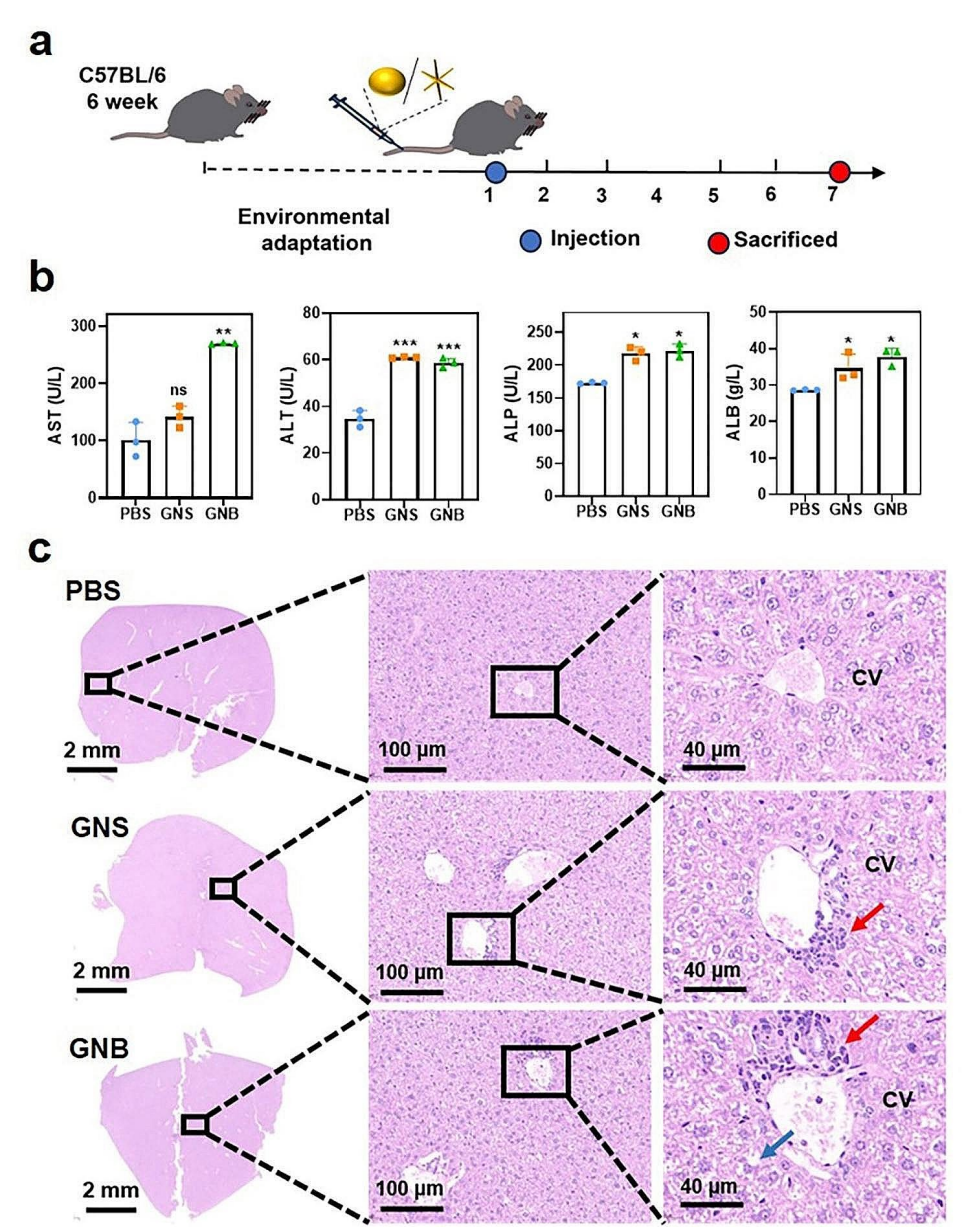
Toxicity of GNSs and GNBs *in vivo.* (**a**) The Schematic diagram of animal experimental design. (**b**) Serum biochemical tests, liver toxicity indicators AST/ALT/ALP, albumin indicator ALB. (**c**) Representative HE staining images of mouse livers. Red arrows point to inflammatory cell infiltration, while the blue arrow points to representative locations of liver cell watery degeneration. CV: central vein. All data are presented as mean ± SD (the dots represent the number of samples). *P < 0.05, **P < 0.01, ***P < 0.001

We then evaluated the in vivo toxicity levels after intravenous injection of GNSs and GNBs, and collected serum and major organs 7 days after injection (Fig. 8a). The body weight of mice was not affected within 7 days of injection of 10 mg/kg GNSS and GNBS (Additional file 7: Fig. S7a), and there was no obvious weight difference in major organs (Additional file 7: Fig. S7b). Distinct variations were observed in blood biochemical indicators (ALT, AST, ALP and ALB). Compared with the control group, liver injury indicators, and serum albumin content largely increased after the injection of GNSs and GNBs, indicating severe liver injury, especially in the liver parenchymal cells with ALB synthesis function (Fig. 8b). AST is mainly present in mitochondria, while ALT is mainly present in the cytoplasm. When liver cells are mildly damaged, ALT is first released into the bloodstream. When liver cells and mitochondria are severely damaged, AST is massively released into the bloodstream. After the injection of GNBs in vivo, the AST signal was much higher than that after the treatment of GNSs, indicating that the mitochondrial damage degree caused by GNBs was more serious than that caused by GNSs, which is consistent with the results on the Hep-orgs. This confirms that the Hep-orgs* in vitro* represents a reliable hepatocyte response compared to the in vivo mice model, with a more mature hepatocyte function and sensitive response than the cellular model.

The distribution, uptake, and metabolism of the material in mice are different from cellular and organoid models [50]. The resistance of models to nanotoxicity increases with their complexity. A larger dose of the nanomaterials needs to be injected for the observation of liver injury. As shown in Fig. 8c, widely visible watery degeneration of liver cells is observed in liver tissue, with liver cells swelling into balloon-like structures, liver sinuses being compressed, and liver cell boundaries becoming unclear. This is consistent with the results we obtained in Hep-orgs in vitro (Fig. 4c), which further proves that GNBs treatment disrupts hepatocyte connectivity and cell membrane integrity. The blue arrows point to the loose cytoplasm, while the red arrows point to the infiltration of inflammatory cells. Exposure to GNSs and GNBs caused an infiltration and accumulation of renal inflammatory cells (Additional file 8: Fig. S8a). The renal function indicator UREA of the GNSs/GNBs group is slightly higher than that of the PBS group, indicating liver and kidney dysfunction, but there are no statistical differences (Additional file 8: Fig. S8b). Similarly, HE staining images of the mouse spleen showed that GNSs and GNBs caused varying degrees of damage to a small amount of central granulocyte aggregation in the spleen (Additional file 9: Fig. S9) and cardiac muscle fiber orientation (Additional file 10: Fig. S10). GNPs injected into the veins of mice are transported through the sinusoidal endothelial cell gaps and captured by liver cells, where they are cleared through the liver and bile. The above results have confirmed that GNSs and GNBs have varying degrees of liver damage* in vivo*, with GNBs exhibiting much more significant toxicity due to nano spikes. This work reminds us that when developing GNPs for biomedical purposes, it is necessary to consider the shape-dependent nanotoxicity and biological effects. More attention should be paid to reducing the shape toxicity of GNPs, material reshaping and surface modification can be employed to comprehensively refine the biological evaluation and to aid the clinical translation of NPs.

## Conclusion

In summary, this work used hepatocytes, hepatocyte organoid models, and mice to evaluate the shape-mediated hepatotoxicity of gold nanomaterials and in vivo. The tip effect of gold nanomaterials plays a crucial role in the cellular toxicity. GNBs exhibit unique nanotoxicity through mitochondrial damage, ROS production, and liver cell metabolism interference. Therefore, it is essential to consider the shape factor of nanomaterials when developing gold nanomaterials for biomedical applications. Compared to the cellular models, Hep-orgs provide more mature liver cell functions and are more sensitive to the cytoskeleton and mitochondrial damage. The organoid platform in vitro offers a rapid and three-dimensional view closest to in vivo conditions for exploring the mechanism of nanomaterials’ hepatotoxicity. Although Hep-orgs recapitulates crucial features of the liver, such as structure, major cell types, and partial functionality, the exact control over the size, precise cellular composition, and ratios of Hep-orgs currently remains challenging. Currently, this work only focused on the biological effects of Au nanomaterials with different shapes, and the depth of research into the differences in the gene and protein expressions during the process is insufficient, which still needs to be further investigated and explored. More complete and multifunctional organoids such as vascularized organoids and immune co-culture organoids still need to be developed for the screening and evaluation of nanoparticles or nanomedicines. Organoid models establish an efficient and economical platform for understanding and investigating the interactions and mechanisms between nanomedicines and the human body systems, thereby providing robust support for drug development and toxicological assessment.

### Electronic supplementary material

Below is the link to the electronic supplementary material.

**Additional file 1: Fig. S1**. (a) Bright field optical images of liver organoids growing in EM at different time periods. (b) The averaged diameters of liver organoids growing in EM at different time periods. (c) HE staining image and ZO-1 IHC image of the liver organoids. (d) Bright field optical images of the liver organoids after 3 days of subculture and resuscitation culture

**Additional file 2: Fig. S2**. (a) DLS-measured size distributions of GNSs and GNBs. (b) Averaged branch length, spike bottom width, and tip radius of GNBs

**Additional file 3: Fig. S3**. Simulated extinction, absorption and scattering cross-sections of (a) GNS and (b) GNB

**Additional file 4: Fig. S4**. CLSM single-layer images showing the accumulation of GNSs and GNBs in the cavity at 12 h

**Additional file 5: Fig. S5**. The effects of GNSs/GNBs on the structural and physiological states of HepG2 cells. (a) CLSM images of cell cytoskeleton after the treatment with GNSs and GNBs. (Green: F-actin; Blue: DAPI) (b) Corresponding fluorescence intensity ratio of nuclear/F-actin in cells. (c) CLSM images of MMP changes in cells after the treatment with GNSs and GNBs. (Red: J-aggregates; Green: Monomer) (d) Ratio of JC-1 green/red fluorescence intensity in cells. (e) CLSM images of ROS production in cells after the treatment with GNSs and GNBs. (f) Corresponding fluorescence intensity of ROS production in cells. All data are presented as mean ± SD (the dots represent the number of samples). *P<0.05, **P<0.01, ***P<0.001

**Additional file 6: Fig. S6**. The AST (a), and ALT (b) levels of HepG2 and Hep-orgs after being treated with GNSs and GNBs (the dots represent the number of samples). *P<0.05, **P<0.01, ***P<0.001

**Additional file 7: Fig. S7**. (a) Body weight variations as a function of the time periods after the injection of GNSs and GNBs. (b) Weight results of heart, liver, spleen, and kidney of mice sacrificed after 7 days (the dots represent the number of samples)

**Additional file 8: Fig. S8**. a) Representative HE staining images of mouse kidneys. The red arrow points to the infiltration and accumulation of inflammatory cells. (b) Serum biochemical tests using UREA indicator (the dots represent the number of samples)

**Additional file 9: Fig. S9**. Representative HE staining images of mouse spleens

**Additional file 10: Fig. S10**. Representative HE staining images of mouse hearts. The green arrows point to the disordered arrangement of cardiac fibers

## Data Availability

The datasets supporting the conclusions of this article are included within the article and its additional files.

## Abbreviations

GNPs: gold nanoparticles
Hep-orgs: hepatocyte organoid models
GNSs: gold nanospheres
GNBs: gold nanobranchs
BP-QDs: black phosphorus quantum dots
ROS: reactive oxygen species
CLSM: confocal laser scanning microscopy
FDTD: finite-difference time-domain
DMEM: Dulbecco’s modified eagle Medium
