# Supplementary material for: Spike structure of gold nanobranches induces hepatotoxicity in mouse hepatocyte organoid models
Source: J Nanobiotechnology. 2024 Mar 5;22:92. doi: 10.1186/s12951-024-02363-1 (PMC10913213; doi:10.1186/s12951-024-02363-1)
Supplement: Supplementary file 1 — Additional file 1: Fig. S1. (a) Bright field optical images of liver organoids growing in EM at different time periods. (b) The averaged diameters of liver organoids growing in EM at different time periods. (c) HE staining image and ZO-1 IHC image of the liver organoids. (d) Bright field optical images of the liver organoids after 3 days of subculture and resuscitation culture [file 12951_2024_2363_MOESM1_ESM.pptx]

## Slide 1
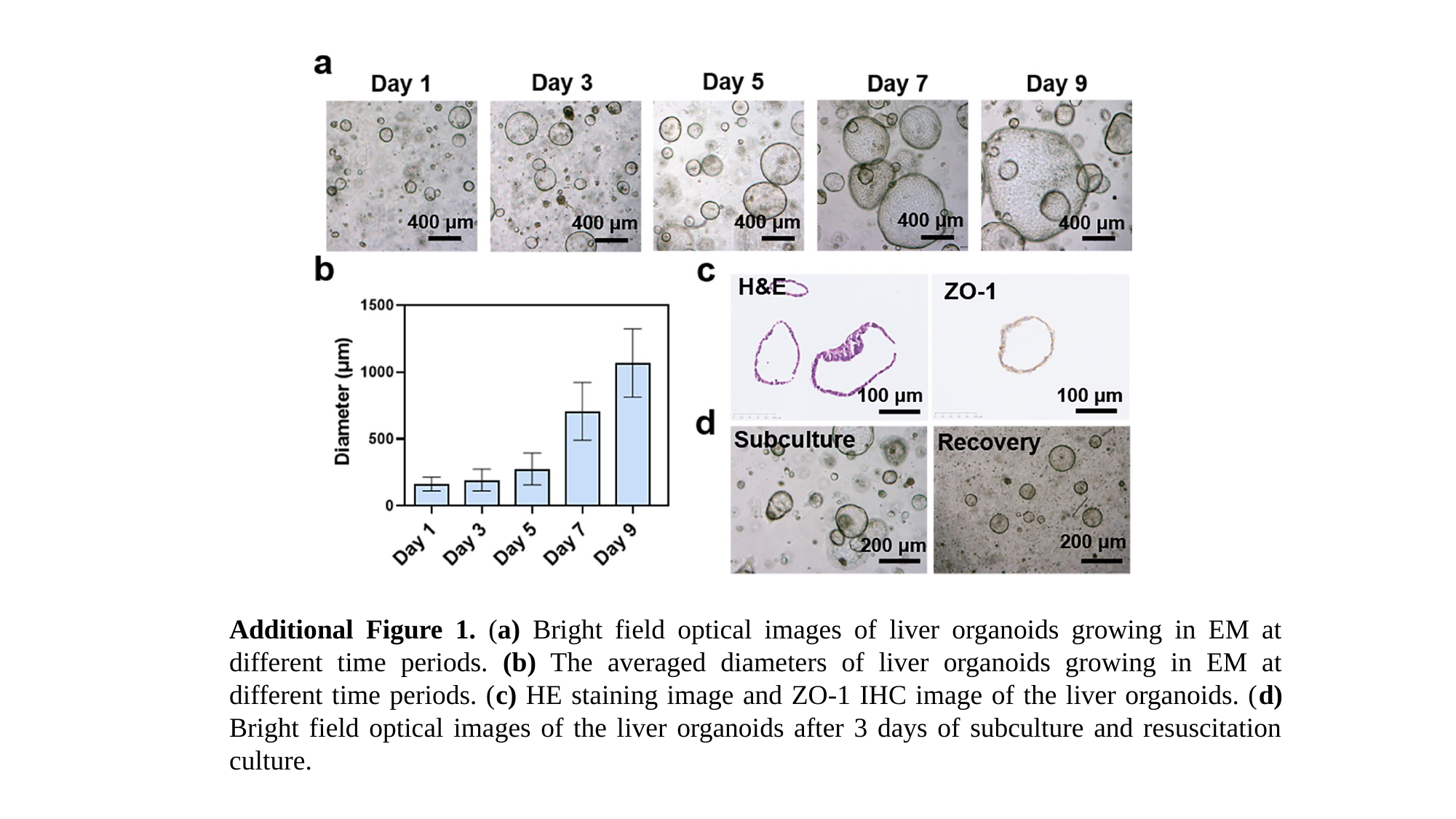

Additional Figure 1. (a) Bright field optical images of liver organoids growing in EM at different time periods. (b) The averaged diameters of liver organoids growing in EM at different time periods. (c) HE staining image and ZO-1 IHC image of the liver organoids. (d) Bright field optical images of the liver organoids after 3 days of subculture and resuscitation culture.
