# Supplementary material for: Spike structure of gold nanobranches induces hepatotoxicity in mouse hepatocyte organoid models
Source: J Nanobiotechnology. 2024 Mar 5;22:92. doi: 10.1186/s12951-024-02363-1 (PMC10913213; doi:10.1186/s12951-024-02363-1)
Supplement: Supplementary file 2 — Additional file 2: Fig. S2. (a) DLS-measured size distributions of GNSs and GNBs. (b) Averaged branch length, spike bottom width, and tip radius of GNBs [file 12951_2024_2363_MOESM2_ESM.pptx]

## Slide 1
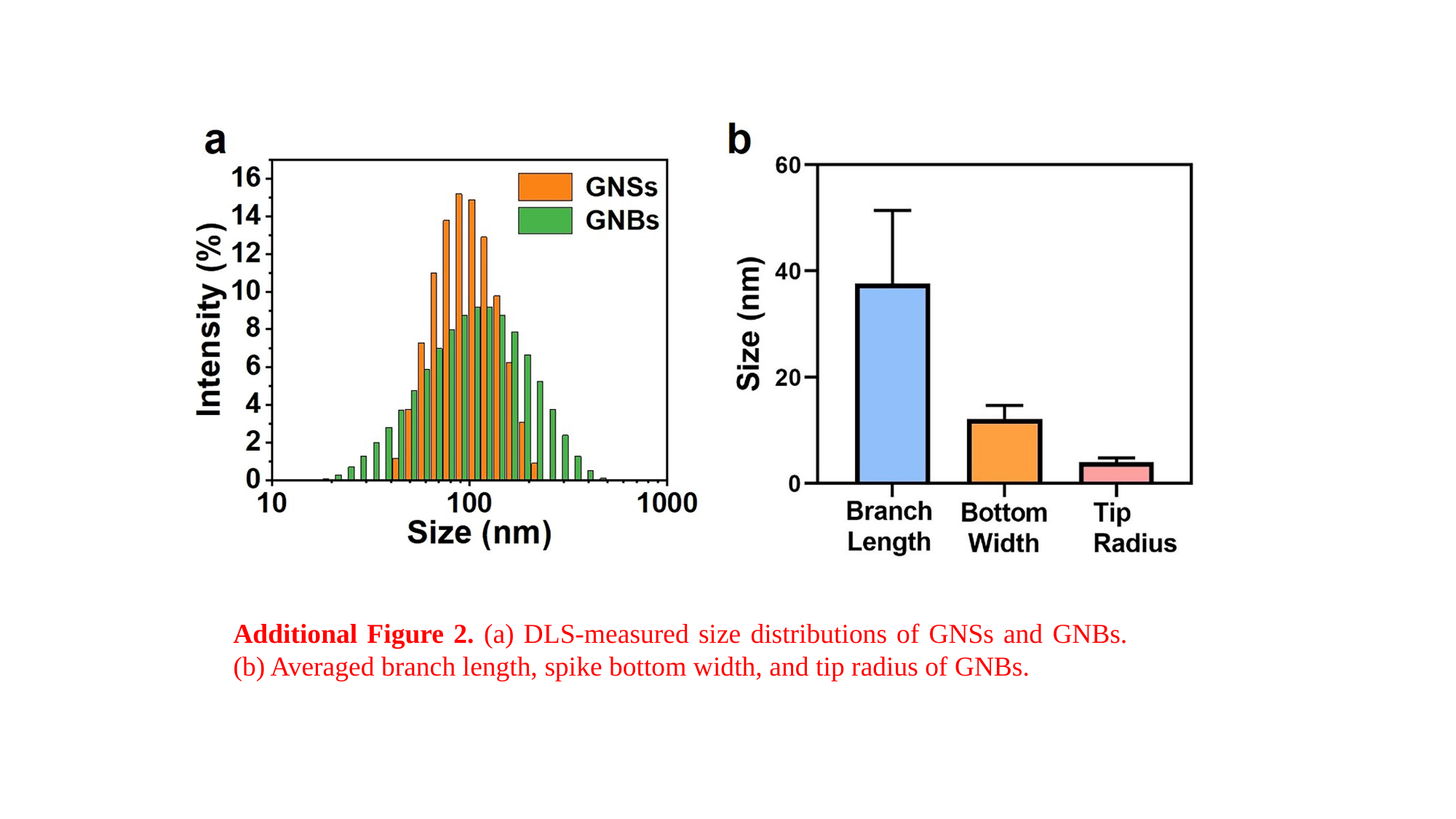

Additional Figure 2. (a) DLS-measured size distributions of GNSs and GNBs. (b) Averaged branch length, spike bottom width, and tip radius of GNBs.
