# Supplementary material for: Spike structure of gold nanobranches induces hepatotoxicity in mouse hepatocyte organoid models
Source: J Nanobiotechnology. 2024 Mar 5;22:92. doi: 10.1186/s12951-024-02363-1 (PMC10913213; doi:10.1186/s12951-024-02363-1)
Supplement: Supplementary file 3 — Additional file 3: Fig. S3. Simulated extinction, absorption and scattering cross-sections of (a) GNS and (b) GNB [file 12951_2024_2363_MOESM3_ESM.pptx]

## Slide 1
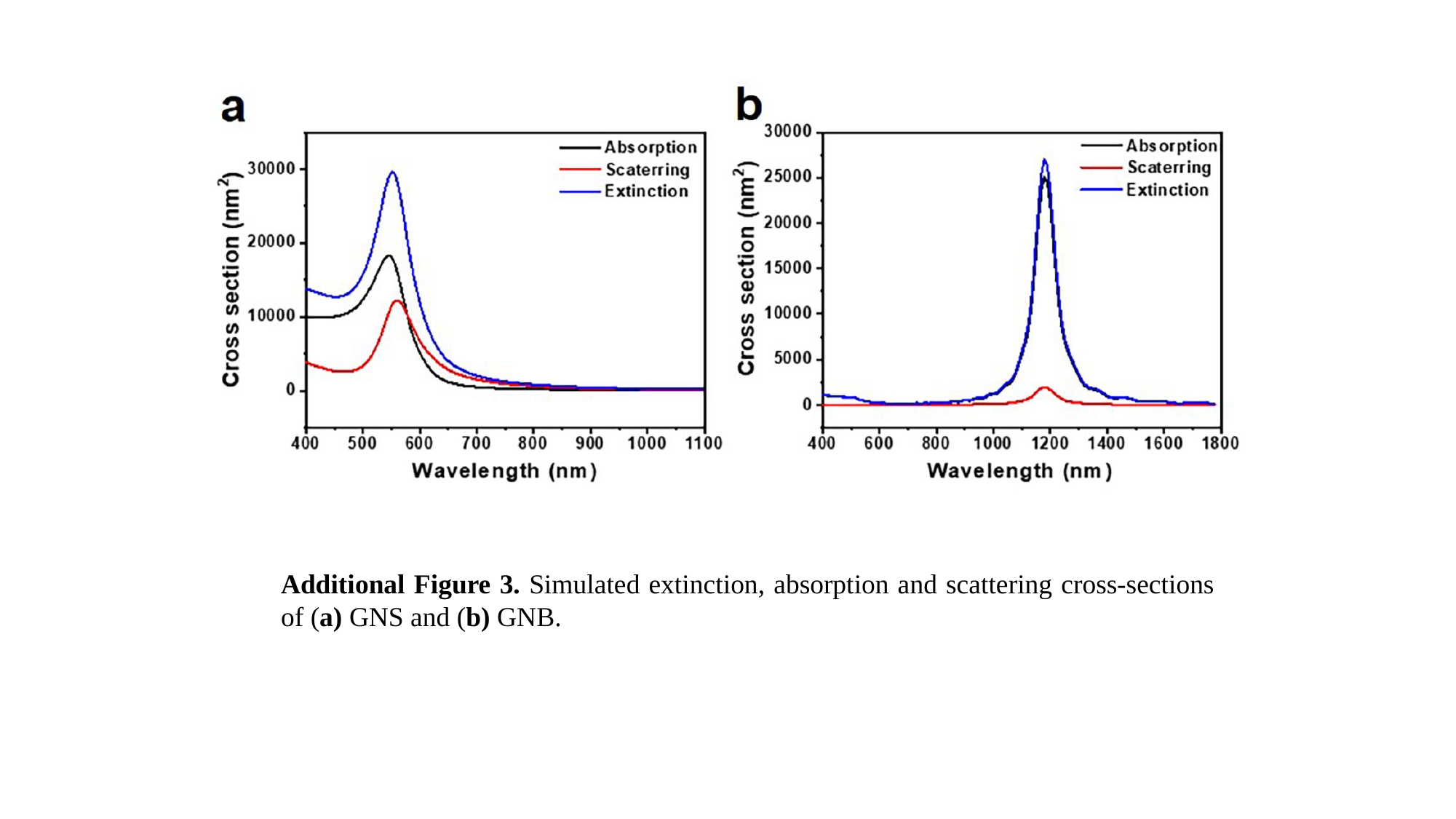

Additional Figure 3. Simulated extinction, absorption and scattering cross-sections of (a) GNS and (b) GNB.
