# Supplementary material for: Spike structure of gold nanobranches induces hepatotoxicity in mouse hepatocyte organoid models
Source: J Nanobiotechnology. 2024 Mar 5;22:92. doi: 10.1186/s12951-024-02363-1 (PMC10913213; doi:10.1186/s12951-024-02363-1)
Supplement: Supplementary file 4 — Additional file 4: Fig. S4. CLSM single-layer images showing the accumulation of GNSs and GNBs in the cavity at 12 h [file 12951_2024_2363_MOESM4_ESM.pptx]

## Slide 1
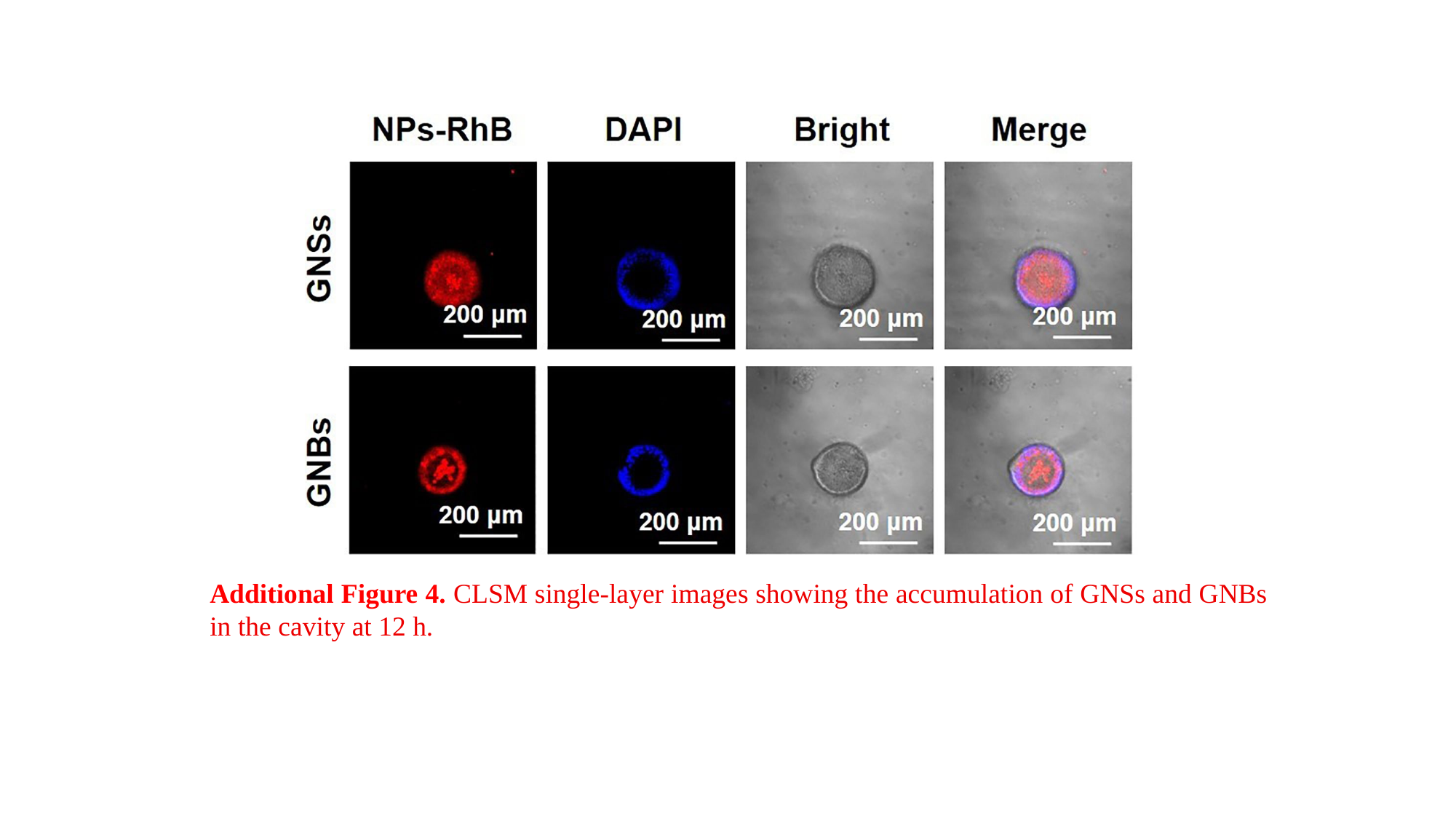

Additional Figure 4. CLSM single-layer images showing the accumulation of GNSs and GNBs in the cavity at 12 h.
