# Supplementary material for: Spike structure of gold nanobranches induces hepatotoxicity in mouse hepatocyte organoid models
Source: J Nanobiotechnology. 2024 Mar 5;22:92. doi: 10.1186/s12951-024-02363-1 (PMC10913213; doi:10.1186/s12951-024-02363-1)
Supplement: Supplementary file 5 — Additional file 5: Fig. S5. The effects of GNSs/GNBs on the structural and physiological states of HepG2 cells. (a) CLSM images of cell cytoskeleton after the treatment with GNSs and GNBs. (Green: F-actin; Blue: DAPI) (b) Corresponding fluorescence intensity ratio of nuclear/F-actin in cells. (c) CLSM images of MMP changes in cells after the treatment with GNSs and GNBs. (Red: J-aggregates; Green: Monomer) (d) Ratio of JC-1 green/red fluorescence intensity in cells. (e) CLSM images of ROS production in cells after the treatment with GNSs and GNBs. (f) Corresponding fluorescence intensity of ROS production in cells. All data are presented as mean ± SD (the dots represent the number of samples). *P<0.05, **P<0.01, ***P<0.001 [file 12951_2024_2363_MOESM5_ESM.pptx]

## Slide 1
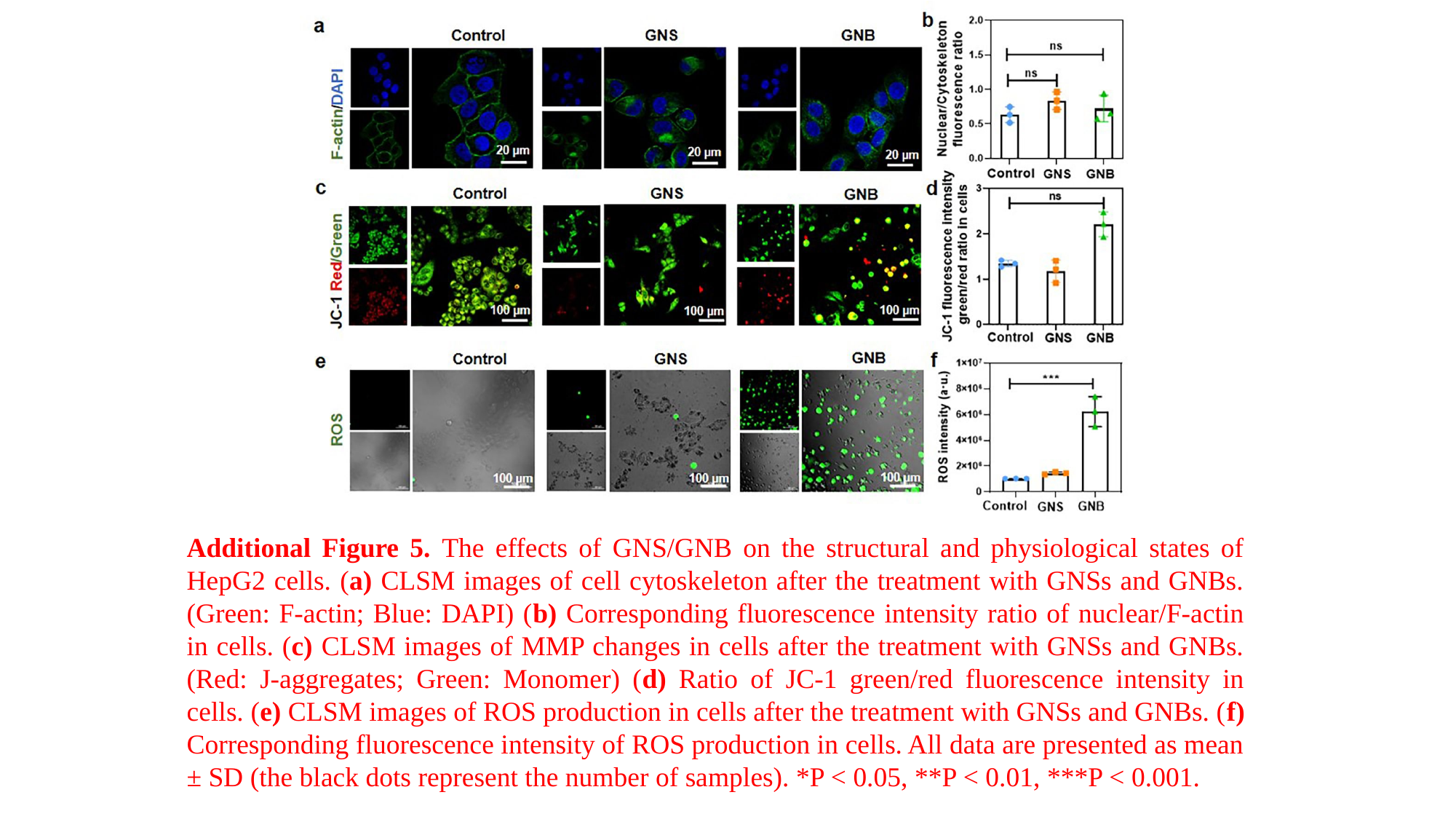

Additional Figure 5. The effects of GNS/GNB on the structural and physiological states of HepG2 cells. (a) CLSM images of cell cytoskeleton after the treatment with GNSs and GNBs. (Green: F-actin; Blue: DAPI) (b) Corresponding fluorescence intensity ratio of nuclear/F-actin in cells. (c) CLSM images of MMP changes in cells after the treatment with GNSs and GNBs. (Red: J-aggregates; Green: Monomer) (d) Ratio of JC-1 green/red fluorescence intensity in cells. (e) CLSM images of ROS production in cells after the treatment with GNSs and GNBs. (f) Corresponding fluorescence intensity of ROS production in cells. All data are presented as mean ± SD (the black dots represent the number of samples). *P < 0.05, **P < 0.01, ***P < 0.001.
