# Supplementary material for: Spike structure of gold nanobranches induces hepatotoxicity in mouse hepatocyte organoid models
Source: J Nanobiotechnology. 2024 Mar 5;22:92. doi: 10.1186/s12951-024-02363-1 (PMC10913213; doi:10.1186/s12951-024-02363-1)
Supplement: Supplementary file 6 — Additional file 6: Fig. S6. The AST (a), and ALT (b) levels of HepG2 and Hep-orgs after being treated with GNSs and GNBs (the dots represent the number of samples). *P<0.05, **P<0.01, ***P<0.001 [file 12951_2024_2363_MOESM6_ESM.pptx]

## Slide 1
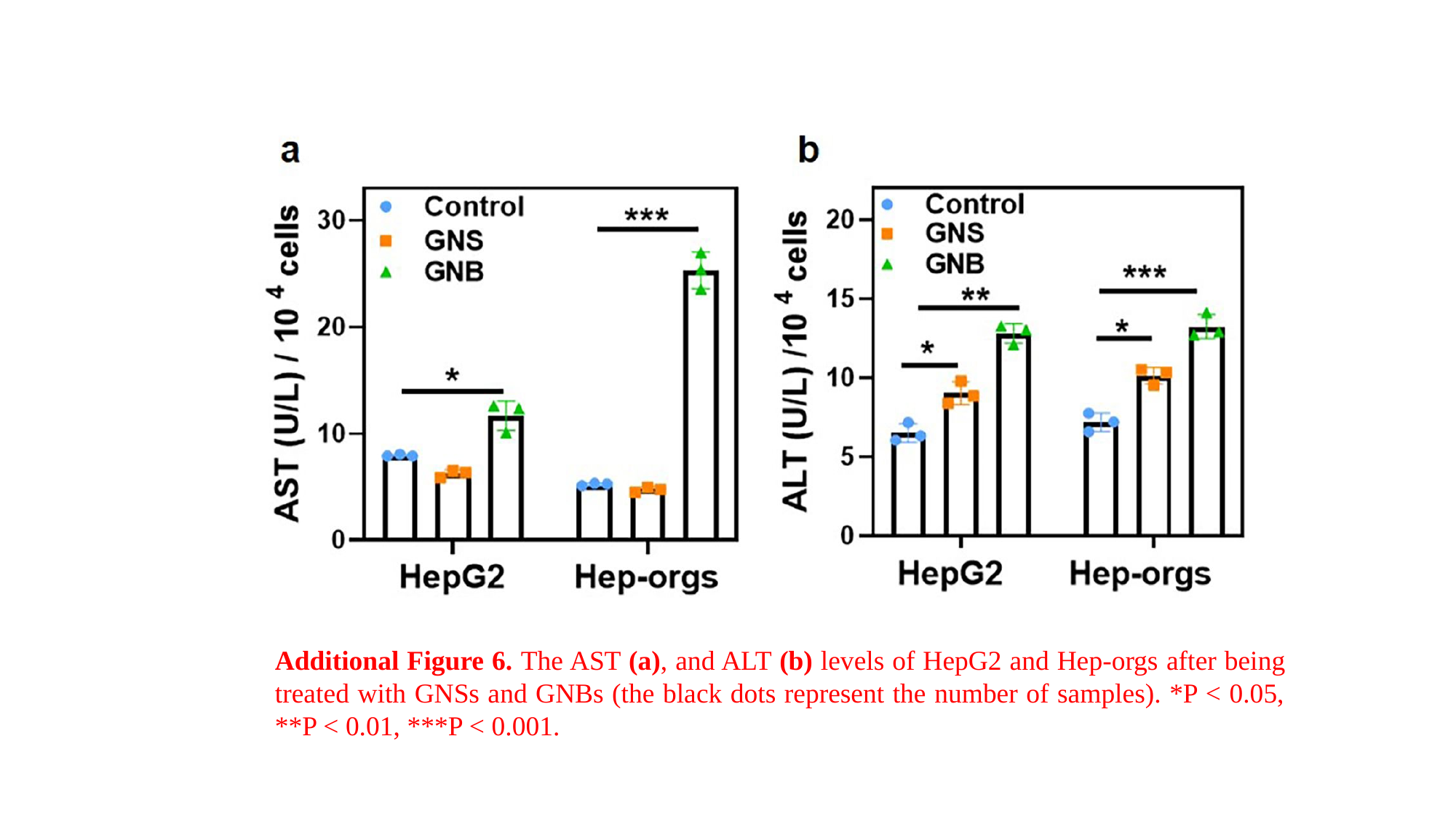

Additional Figure 6. The AST (a), and ALT (b) levels of HepG2 and Hep-orgs after being treated with GNSs and GNBs (the black dots represent the number of samples). *P < 0.05, **P < 0.01, ***P < 0.001.
