# Supplementary material for: Spike structure of gold nanobranches induces hepatotoxicity in mouse hepatocyte organoid models
Source: J Nanobiotechnology. 2024 Mar 5;22:92. doi: 10.1186/s12951-024-02363-1 (PMC10913213; doi:10.1186/s12951-024-02363-1)
Supplement: Supplementary file 7 — Additional file 7: Fig. S7. (a) Body weight variations as a function of the time periods after the injection of GNSs and GNBs. (b) Weight results of heart, liver, spleen, and kidney of mice sacrificed after 7 days (the dots represent the number of samples) [file 12951_2024_2363_MOESM7_ESM.pptx]

## Slide 1
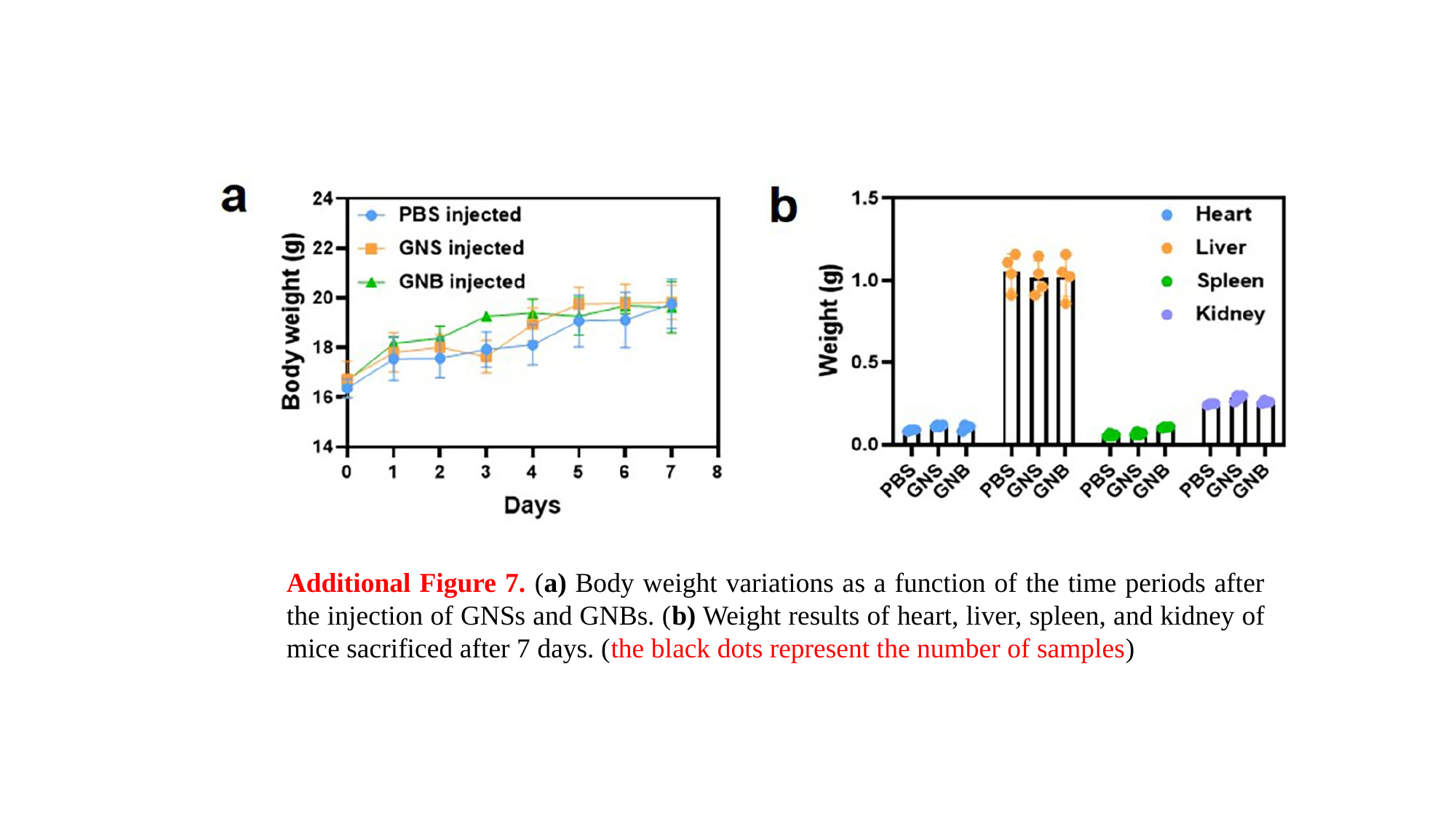

Additional Figure 7. (a) Body weight variations as a function of the time periods after the injection of GNSs and GNBs. (b) Weight results of heart, liver, spleen, and kidney of mice sacrificed after 7 days. (the black dots represent the number of samples)
