# Supplementary material for: Spike structure of gold nanobranches induces hepatotoxicity in mouse hepatocyte organoid models
Source: J Nanobiotechnology. 2024 Mar 5;22:92. doi: 10.1186/s12951-024-02363-1 (PMC10913213; doi:10.1186/s12951-024-02363-1)
Supplement: Supplementary file 8 — Additional file 8: Fig. S8. a) Representative HE staining images of mouse kidneys. The red arrow points to the infiltration and accumulation of inflammatory cells. (b) Serum biochemical tests using UREA indicator (the dots represent the number of samples) [file 12951_2024_2363_MOESM8_ESM.pptx]

## Slide 1
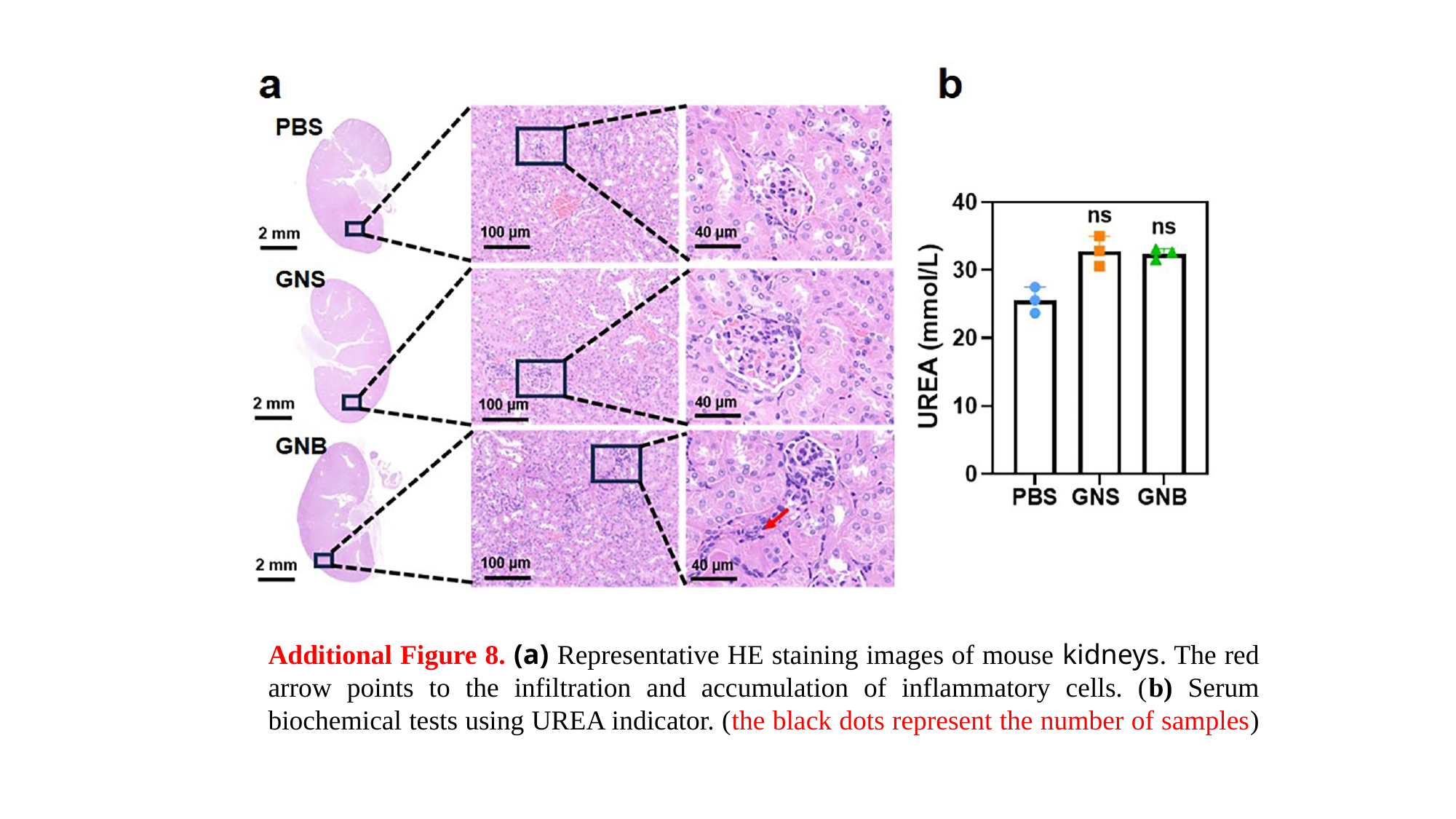

Additional Figure 8. (a) Representative HE staining images of mouse kidneys. The red arrow points to the infiltration and accumulation of inflammatory cells. (b) Serum biochemical tests using UREA indicator. (the black dots represent the number of samples)
