# Supplementary figures and images for: Spike structure of gold nanobranches induces hepatotoxicity in mouse hepatocyte organoid models
Source: J Nanobiotechnology. 2024 Mar 5;22:92. doi: 10.1186/s12951-024-02363-1 (PMC10913213; doi:10.1186/s12951-024-02363-1)

## Slide 1
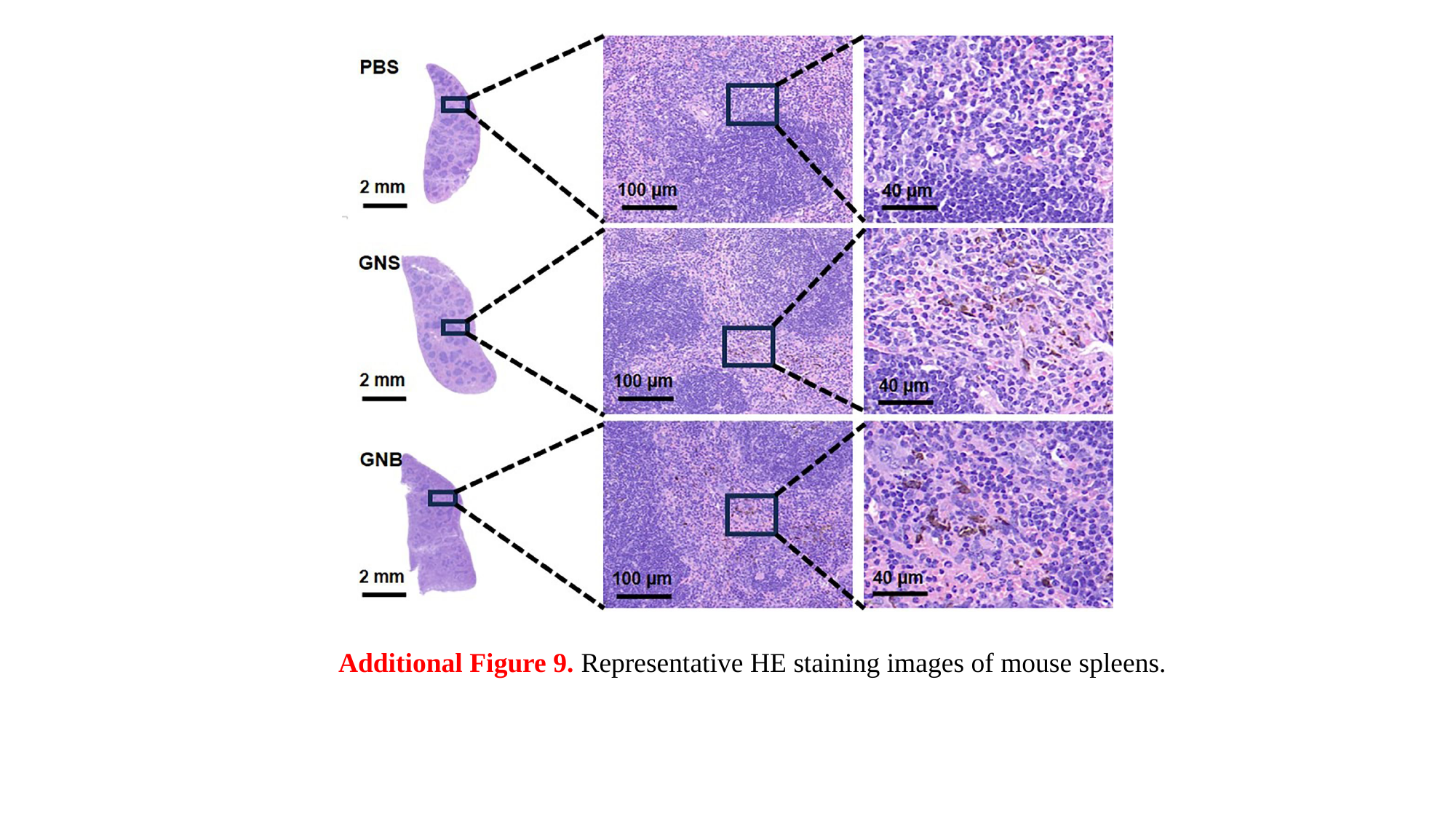

Additional Figure 9. Representative HE staining images of mouse spleens.

Supplement: Supplementary file 9 — Additional file 9: Fig. S9. Representative HE staining images of mouse spleens [file 12951_2024_2363_MOESM9_ESM.pptx]
