# Supplementary material for: Spike structure of gold nanobranches induces hepatotoxicity in mouse hepatocyte organoid models
Source: J Nanobiotechnology. 2024 Mar 5;22:92. doi: 10.1186/s12951-024-02363-1 (PMC10913213; doi:10.1186/s12951-024-02363-1)
Supplement: Supplementary file 10 — Additional file 10: Fig. S10. Representative HE staining images of mouse hearts. The green arrows point to the disordered arrangement of cardiac fibers [file 12951_2024_2363_MOESM10_ESM.pptx]

## Slide 1
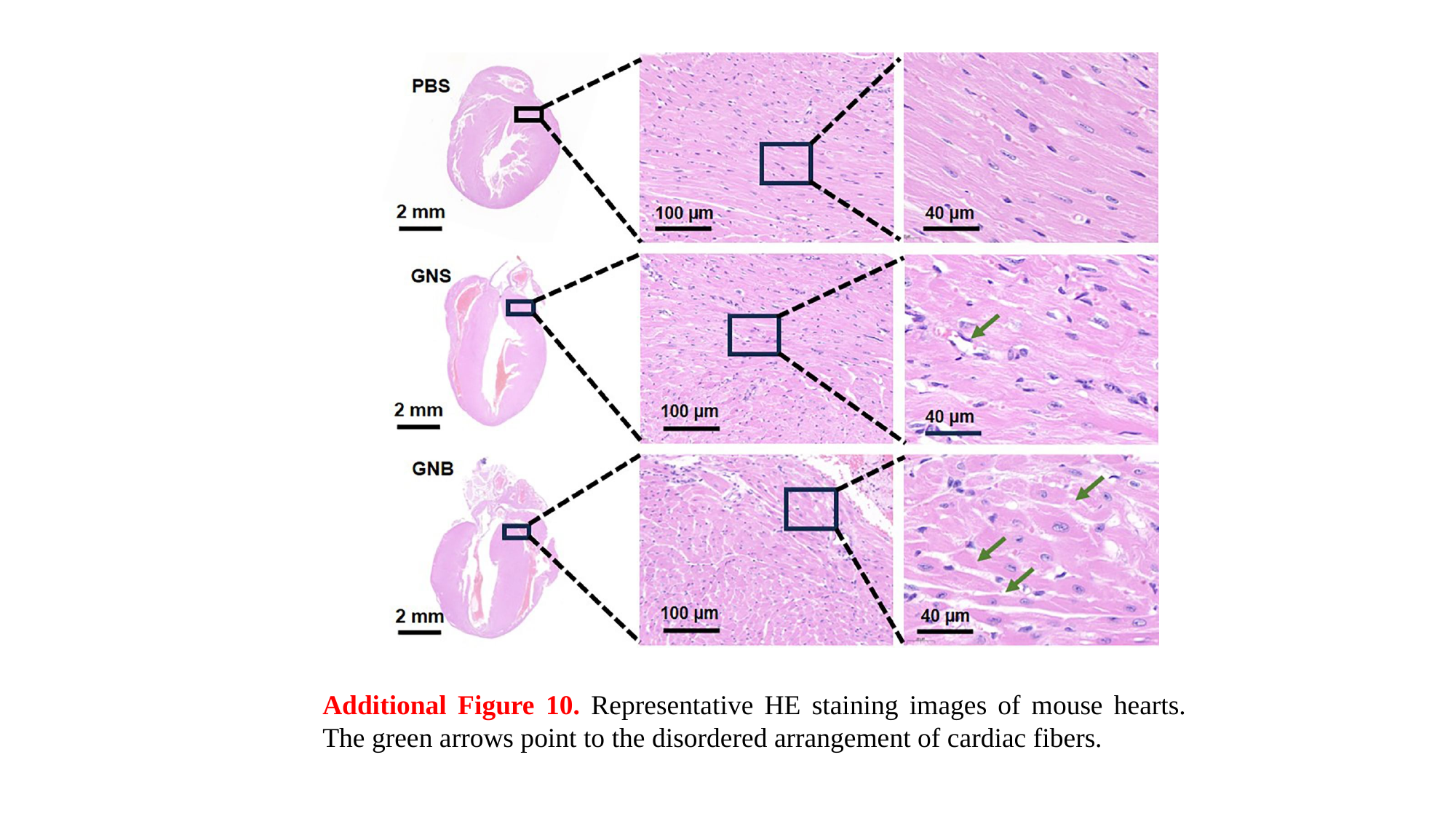

Additional Figure 10. Representative HE staining images of mouse hearts. The green arrows point to the disordered arrangement of cardiac fibers.
